# Structure–Thermoelectric Property Relations of the Nowotny Chimney–Ladder Phase Fe_2_Ge_3_

**DOI:** 10.1021/acs.chemmater.6c00878

**Published:** 2026-08-17

**Authors:** Oleksandr Cherniushok, Illia Lukashchuk, Kacper Pryga, Raisa Tahiyah, Taras Parashchuk, Yaroslav Tokaychuk, Lev Akselrud, Bartlomiej Wiendlocha, Roman Gladyshevskii, Yuri Grin, Alexandra Zevalkink, Krzysztof T. Wojciechowski

**Affiliations:** † Thermoelectric Research Laboratory, Department of Inorganic Chemistry, Faculty of Materials Science and Ceramics, 513364 AGH University of Krakow, Mickiewicza Ave. 30, 30-059 Krakow, Poland; ‡ Faculty of Physics and Applied Computer Science, AGH University of Krakow, Mickiewicza Ave. 30, 30-059 Krakow, Poland; § Department of Chemical Engineering and Materials Science, 3078 Michigan State University, East Lansing, Michigan 48824, United States; ∥ Department of Inorganic Chemistry, 112865 Ivan Franko National University of Lviv, Kyryla i Mefodiya St. 6, 79005 Lviv, Ukraine; ⊥ 28270 Max-Planck-Institut Für Chemische Physik Fester Stoffe, Nöthnitzer Str. 40, 01187 Dresden, Germany

## Abstract

The Nowotny chimney–ladder (NCL) phase in the Fe–Ge system near Fe_2_Ge_3_ composition is a promising thermoelectric material owing to its characteristic coexistence of degenerate electronic transport and strongly suppressed lattice thermal conductivity, yet the structural and compositional origins of this behavior remain incompletely understood. Synchrotron diffraction shows that samples prepared with nominal Fe_2_Ge_3_ composition crystallize as an incommensurately modulated NCL phase close to FeGe_1.52_. From the superspace description, we derive and refine for the first time a three-dimensional commensurate approximant, Fe_27_Ge_41_ (*tP*272). Chemical-bonding analysis based on the model of Fe_2_Ge_3_ reveals a three-dimensional framework of heteroatomic multiatomic Fe–Ge interactions without homoatomic Fe–Fe or Ge–Ge bonds. DFT calculations for the Fe_27_Ge_41_ approximant show that slight Ge excess in comparison with Fe_2_Ge_3_ shifts the Fermi level into the conduction band, yielding a nominal carrier concentration of ∼10^21^ cm^–3^, consistent with Hall measurements, and explaining the degenerate *n*-type behavior without extrinsic donors. The electronic structure is characterized by a high density-of-states effective mass (∼11 *m*_e_), arising from convergence of flat conduction bands near the Fermi level. Despite high elastic moduli and a moderate Grüneisen parameter, the lattice thermal conductivity is low (∼1.8 W m^–1^ K^–1^ at 300 K), corresponding to an estimated phonon mean free path of approximately 8 Å. The failure of the Debye–Callaway model to reproduce this value indicates additional phonon scattering, which may be related to the incommensurate structure and bonding inhomogeneity. Overall, the incommensurate Fe–Ge NCL phase combines degenerate electronic transport with phonon-glass-like thermal behavior.

## Graphical abstract

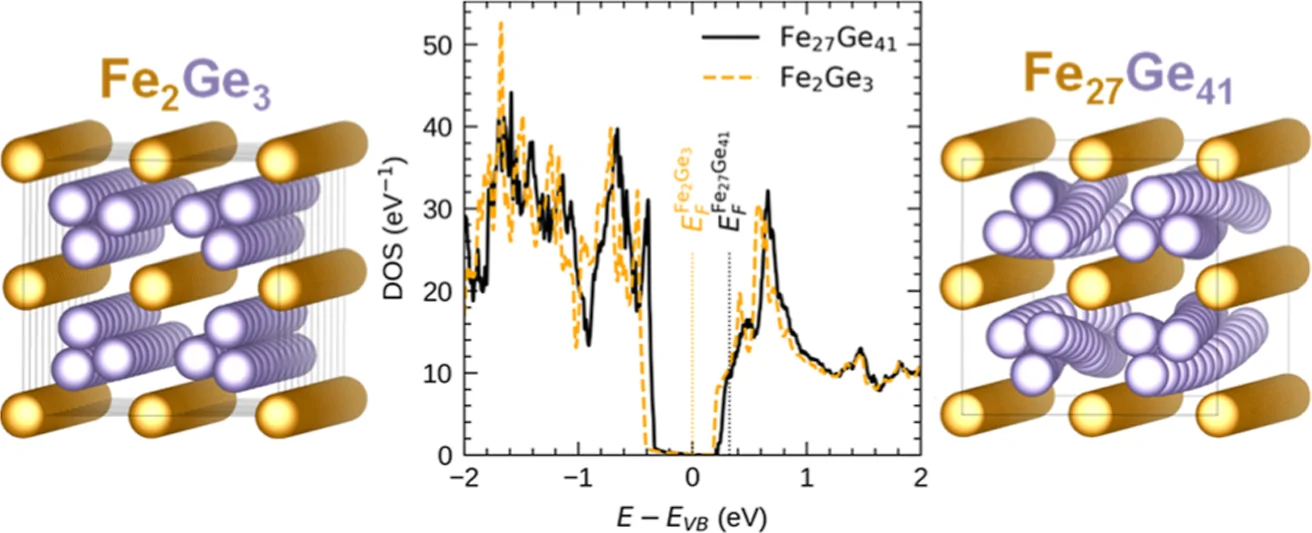

## 1 Introduction

Thermoelectric materials are capable of direct energy conversion between thermal and electrical forms, a technology that has attracted increased attention in recent years for waste heat recovery and solid-state cooling.^1,2^ The performance of these materials is dictated by the dimensionless figure of merit ZT, defined as ZT = *S* ^2^σ*T*/κ, where *S* is the Seebeck coefficient, σ is the electrical conductivity, *T* is the absolute temperature, and κ is the total thermal conductivity.^3,4^ The primary challenge in optimizing ZT is the strong interdependence of its parameters,^5^ since *S*, σ, and the electronic thermal conductivity κ_e_ are all governed by the carrier concentration. This has led to the search for materials that can optimize the balance between these transport properties.^6–8^ The Nowotny chimney–ladder (NCL) phases are particularly significant in this context because their unique structural architecture provides an intrinsic solution to this problem, offering a platform to realize a state where electronic and thermal transport are independently controlled.^9–15^

NCL phases are a class of intermetallic compounds with a distinctive crystal structure, which was traditionally represented as two interpenetrating sublattices.^9,16–19^ The first is a tetragonal “chimney” of transition metal atoms, while the second is a spiraling “ladder” of main group atoms embedded within the chimney.^10,11^ The stability and properties of the majority of NCL phases are closely tied to their valence electron count (VEC), following the empirical “14-electron rule” where the total number of valence electrons per transition metal atom is approximately 14.^13^

The Fe–Ge binary system hosts an NCL type compound near the Fe_2_Ge_3_ composition that can crystallize either as a commensurate or incommensurate phase depending on stoichiometry and synthesis route. Early work by Li et al.^20^ identified a new Fe-based NCL phase in a polycrystalline sample with composition FeGe_1.52_, featuring satellite reflections in electron diffraction typical of incommensurate modulations. Le Tonquesse et al.^21^ refined this structure within the (3 + 1)-dimensional superspace group *I*4_1_/*amd*(00γ)­00*ss* with basic lattice parameters *a* = 5.5924(3) Å, *c*_Fe_ = 4.4948(3) Å, *c*_Ge_ = 2.959(1) Å, γ = 1.5190(1), and a modulation vector *q* = (0, 0, 1.5191(1)). Note that in an incommensurate lattice, γ is defined by the ratio of *c*_Fe_/*c*_Ge_. Further, the value of γ directly controls the stoichiometry of the phase, which can be written as FeGe_γ_.

In contrast to the polycrystalline samples described above, single crystals^22,23^ yielded the commensurate Fe_2_Ge_3_ phase (i.e., γ = 1.5) with a tetragonal structure (space group *P*4̅*c*2, No. 116) and lattice parameters *a* = 5.5995(6) Å, *c* = 8.9531(14) Å.^23^ Here, the Fe and Ge substructures are perfectly matched along *c*, giving rise to a commensurate NCL framework of Fe chimneys enclosing Ge ladders. No superlattice reflections or modulations were observed. The commensurate Fe_2_Ge_3_ structure satisfies the 14-electron rule and is predicted to exhibit a narrow pseudogap that stabilizes the ideal framework. However, transport measurements in both single-crystalline and polycrystalline samples report a negative Seebeck coefficient, indicating that the Fermi level in real materials is shifted away from the pseudogap, consistent with a deviation from the ideal VEC. Commensurate Fe_2_Ge_3_ single crystals are narrow-gap semiconductors with a reported band gap below 0.1 eV,^22,24^ whereas polycrystalline samples exhibit an apparent transport gap of ∼0.03 eV.^23^ This value, however, as derived from the Arrhenius activation law applied to electrical resistivity in the strongly degenerated regime, is not an accurate measure of the bandgap.

The material is noted for its remarkably low and “glass-like” thermal conductivity, with reported values of below 1.0 W m^–1^ K^–1^ for incommensurate polycrystalline samples at 373 K and 1.9 W m^–1^ K^–1^ at 300 K for commensurate single crystals.^23,25^ This low thermal conductivity is attributed to structural complexity as well as specific phonon-level mechanisms, including low-energy optical phonons, Einstein modes, “twisting modes”, and “anticrossings”, which scatter heat-carrying acoustic phonons.^25^ The highest reported ZT for a polycrystalline sample is 0.57 at 600–633 K, which is supported by a large power factor of 1.90 mW m^–1^ K^–2^.^25^ Theoretical calculations based on an idealized Ru_2_Sn_3_-type Fe_2_Ge_3_ model have predicted a potential ZT exceeding unity, with an ultrahigh peak value of ∼2.4 suggested upon optimization of the VEC.^25^

To date, theoretical descriptions of the electronic properties of the Fe_2_Ge_3_ system have been based almost exclusively on the commensurate Ru_2_Sn_3_-type structural model.^22,24^ While this approximation has provided useful qualitative insight, it is not sufficient to capture the electronic ground state of incommensurately modulated structure close to FeGe_1.52_ for polycrystalline samples. As a result, the transport behavior of these materials—particularly the origin of their degenerate carrier concentrations and suppressed lattice thermal conductivity—has remained difficult to rationalize within a chemically consistent framework. The primary roadblock has been the lack of a structural description that simultaneously reflects the incommensurate nature of the NCL framework and remains compatible with first-principles calculations.

Motivated by these bottlenecks in the understanding, the present work addresses the lack of a structural model suitable for quantitative electronic-structure and transport calculations in Fe–Ge Nowotny phases. Starting from an accurate crystallographic description of the incommensurately modulated structure observed in samples with nominal Fe_2_Ge_3_ composition, we construct and refine a fully three-dimensional commensurate approximant, Fe_27_Ge_41_. This approximant captures the essential structural features of the incommensurate chimney–ladder framework while remaining compatible with first-principles methods, thereby providing a consistent basis for examining the role of stoichiometry, electronic band structure, and lattice thermal transport in the Fe–Ge system.

## 2 Experimental and Calculational Details

### 2.1 Materials and Synthesis

Samples with the nominal compositions of Fe_2_Ge_3_ were prepared by melting high-purity Fe (powder, 99.99%) and Ge (shot, 99.999%) in quartz containers evacuated to a residual pressure of 10^–4^ mBar. The total mass of every sample was 10 g. The ampules with the stoichiometric mixtures of elements were heated up to 1473 K at the rate of 108 K/h; kept at this temperature for 10 h; and cooled down to room temperature with the same rate. To obtain homogeneous samples, obtained ingots were crushed and milled into fine powders, compacted using a spark plasma sintering (SPS) at 773 K and 60 MPa for 100 min, then heated up in evacuated quartz ampules up to 773 K with the rate of 250 K/h, annealed at this temperature for 300 h, and quenched in cold water without breaking the containers.

### 2.2 Sintering

After annealing, the material was milled into fine powder using a planetary ball mill and subsequently densified by SPS at 773 K for 120 min. The temperature of sintering has been selected due to the known decomposition of this phase above 853 K.^26^ The process was conducted in a 10 mm diameter graphite die under an axial compressive stress of 60 MPa in an argon atmosphere. The heating and cooling rates were set to 70 and 20 K/min, respectively. The relatively long sintering duration was expected to improve the sample homogeneity. The resulting pellet, with a diameter of 10 mm and a height of ∼12 mm, was polished for transport property measurements. Its density reached approximately 88% of the theoretical value.

### 2.3 Structural, Microstructural, and Thermal Analyses

High-resolution synchrotron X-ray diffraction (XRD) measurements were performed at beamline ID31 at the European Synchrotron Radiation Facility (ESRF). The annealed sample powder was loaded into cylindrical slots (approximately 1 mm thickness) held between Kapton windows in a high-throughput sample holder and measured in transmission geometry with an incident X-ray energy of 75.051 keV (λ = 0.16520 Å). Measured intensities were collected using a Pilatus CdTe 2 M detector (1679 × 1475 pixels, 172 × 172 μm^2^ each) positioned with the incident beam in the corner of the detector. The sample-to-detector distance was approximately 1.5 m for the high-resolution measurements. Background measurements for the empty windows were measured and subtracted. NIST SRM 660b (LaB_6_) was used for geometry calibration performed with the software pyFAI,^27^ followed by image integration including a flat-field, geometry, solid-angle, and polarization corrections. Rietveld refinement of the crystal structure was carried out using the WinCSD program package.^28^

Thermal analysis of the synthesized materials was carried out by using a differential scanning calorimeter (NETZSCH DSC 404 F3 Pegasus). Approximately 15 mg of the annealed sample was placed in an aluminum crucible with a lid. The measurements were conducted at a heating rate of 10 K/min under helium flow. DSC analysis was employed to identify phase transitions and evaluate the thermal stability of the materials.

For microstructural and compositional studies, the samples were first consolidated in the SPS and then were embedded in resin, mechanically ground, and polished using a 0.1 μm diamond slurry. Prior to examination, a thin conductive graphite coating was sputtered onto the surface. Microstructural homogeneity and local chemical composition were investigated using a ThermoFisher Scientific Scios 2 scanning electron microscope equipped with standardless energy-dispersive X-ray spectroscopy (EDS).

### 2.4 Crystal Structure Study

Observed peaks on the synchrotron XRD pattern were successfully indexed using a composite lattice with the superspace group *I*4_1_/*amd*(00γ)­00*ss* and the cell parameters: *a* = 5.59351(1), *c*_Fe_ = 4.49658(2), *c*_Ge_ = 2.96067(1) Å. Here, *c*_Fe_ and *c*_Ge_ denotes the parameters of the Fe and Ge subcells, which are commensurate in the crystallographic directions [100] and [010] (*a* and *b*), but incommensurate along the crystallographic direction [001] (*c*). The atomic positions in the Fe substructure were determined using the reflections *hkl*0 within the space group *I*4_1_/*amd*, whereas the atomic positions in the Ge substructure were obtained from the reflections *hk*0*m* within the same space group. The obtained results were subsequently combined into a composite model. For the determination of the atomic coordinates the direct-phase determination technique, implemented into WinCSD program package^28^ was used.

Atomic positions obtained from the structure solution of both substructures were used as a starting model for the Rietveld refinement in the superspace group *I*4_1_/*amd*(00γ)­00*ss*. An approximant structure was derived from the incommensurately modulated model. Structural parameters were refined by the Rietveld method and then standardized with STRUCTURE TIDY program.^29^ All calculations, including profile fitting, determination and refinement of the cell parameters, atomic coordinates, displacement parameters, were performed by using WinCSD program package.^28^

### 2.5 Electrical and Thermal Transport Properties

The Seebeck coefficient (*S*) and electrical conductivity (σ) were measured using a commercial NETZSCH SBA 458 Nemesis system under argon flow in the temperature range of 298–773 K. Thermal diffusivity (α_D_) was determined with a NETZSCH LFA 457 MicroFlash instrument. To minimize errors associated with sample emissivity and laser reflection from the shiny pellet surface, the specimen was spray-coated with a thin graphite layer prior to measurement. The thermal conductivity (κ) was calculated according to κ = ρ*C*_p_α_D_, where ρ is the density determined by the Archimedes method from SPS-prepared discs, and *C*_p_ represents the specific heat capacity. *C*_p_ values were estimated from the Dulong–Petit limit and subsequently applied in the calculation of κ. The measurement uncertainties were estimated at 7% for *S*, 5% for σ, and 3% for α_D_, resulting in an overall uncertainty of approximately 20% for the thermoelectric figure of merit ZT.^30^ The spatial distribution of the Seebeck coefficient across the sample surface was examined using a scanning thermoelectric microprobe (SThM) with a spatial resolution of 1 μm. Hall effect measurements were performed by the Van der Pauw method using a Linseis HCS-1 system under constant electric and magnetic fields (*H* = 0.7 T), with a current of 50 mA applied to the sample. The uncertainty of the Hall measurements was estimated at ∼10%.

### 2.6 Elastic Moduli and Speed of Sound Measurements

The longitudinal and transverse sound velocities were determined at 298 K by using an Olympus Epoch 650 ultrasonic flaw detector. The temperature-dependent elastic moduli were measured from 298 to 723 K on a cylindrical sample using resonant ultrasound spectroscopy (RUS) in a custom high-temperature setup with flowing argon atmosphere from Alamo Creek Engineering.^31^ The temperature was increased in 25 K increments with an equilibration period of 15 min at each step to ensure thermal stability. At each temperature, a slow sinusoidal frequency sweep from 250 to 750 Hz was applied. The spectra were analyzed using the RUScal software to match calculated and observed resonance frequencies through iterative fitting allowing extraction of the elastic moduli and their temperature dependence.^32^

### 2.7 Electronic Structure and Transport Calculations

Ab initio calculations were performed using full-potential augmented-plane-wave plus local orbitals method as implemented in the WIEN2k package.^33,34^ Relaxation of atomic coordinates was performed based on the refined approximant model Fe_27_Ge_41_ (experimental lattice parameters as given in Table 1, unit cell contains 272 atoms, i.e. 4 f.u.) with force convergence limits of 0.5 mRy/a.u., and using the generalized gradient approximation with the Perdew–Burke–Ernzerhof (PBE) exchange–correlation functional.^36^ Further calculations of the electronic structure and transport properties were done using the modified Becke–Johnson (mBJ) semilocal exchange–correlation potential.^35^ For a self-consistent field (SCF) cycle, a dense *k*-point sampling was utilized with a 13 × 13 × 1 grid and total energy convergence limit of 10^–4^ Ry. Densities of states and eigenvalues for the transport properties studies were calculated on a denser, 65 × 65 × 3 *k*-point grid, and the calculations were done using BoltzTraP code.^37^ To indicate the importance of structural complexity of NCL phases, calculations were also performed for the commensurate Fe_2_Ge_3_ phase from ref 22 as the starting information. Here, the atomic positions were also optimized in the same way as for Fe_27_Ge_41_ model, and further computational details for the electronic structure calculation in the case of Fe_2_Ge_3_ remain unchanged with respect to the parameters used for Fe_27_Ge_41_ apart from the difference in *k*-point grid as 50 × 50 × 31 was utilized in this case. Convergence tests with respect to the *k*-point grid were performed in the case of both Fe_27_Ge_41_ and Fe_2_Ge_3_ to confirm that the *k*-space sampling is suitable (see Figure S1). Electronic structure and transport calculations for the Fe_27_Ge_41_ approximant were performed in the scalar-relativistic manner, as the analysis for the commensurate Fe_2_Ge_3_ phase indicated negligible effect of the spin–orbit coupling (Figure S4 in the Supporting Information).

**Table 1:** Crystallographic Data for the Incommensurately Modulated Nowotny Chimney–Ladder Phase FeGe_1.52_ and the Commensurate Fe_27_Ge_41_ Approximant

phase composition	FeGe_1.52_	Fe_27_Ge_41_
(super)space group	*I*4_1_ */amd*(00γ)00*ss*	*P*4̅*n*2
unit-cell parameters	*a* = 5.59351(1) Å	*a* = 5.59328(2) Å
	*c*_Fe_ = 4.49658(2) Å	*c* = 121.3826(7) Å
	*c*_Ge_ = 2.96067(1) Å	
cell volume *V*, Å^3^	140.686(1)	3797.43(2)
formula units per cell *Z*	4	4
modulation wave vector γ (*c*_Fe_/*c*_Ge_)	1.51877(2) [001]	-
radiation type; wavelength λ, Å	synchrotron; 0.16520	synchrotron; 0.16520
2θ range, °; 2θ step, °	1.00–13.55; 0.001	1.00–13.55; 0.001
ranges of *h*, *k*, *l*, (*m*)	0 ÷ 8, 0 ÷ 5, 0 ÷ 8, −2 ÷ 2	0 ÷ 5, 0 ÷ 8, 0 ÷ 178
density ρ_X_, g cm^–3^	7.84	7.84
mode of refinement	full profile	full profile
reliability factors: *R*_I_, *R*_p_, goodness of fit	0.1197, 0.1449, 1.04	0.0713, 0.1464, 0.99

### 2.8 Chemical Bonding

Analysis of chemical bonding in position space was performed utilizing the electron localizability approach.^38^ The calculations were performed with the FPLO software (LDA, Perdew–Wang parametrization^39,40^ ). The electron density (ED) and the electron localizability indicator (ELI-D) were calculated within the FPLO framework.^41^ The topological features of the computed distributions of ED and ELI-D were analyzed with the program DGrid.^42^ To obtain information about the interacting atoms, the electron density was first integrated within atomic basins, i.e., spatial regions confined by zero-flux surfaces in the gradient field of ED. This technique represents the procedure proposed in the quantum theory of atoms in molecules (QTAIM^43^) and provides effective electron populations for the QTAIM atoms. Further information about the bonding between atoms is obtained from a combined analysis of ED and ELI-D.^38^ For investigation of chemical bonding, the commensurate model of Fe_2_Ge_3_ (Ru_2_Sn_3_-type, space group *P*4̅*c*2, *a* = 5.5848 Å, *c* = 8.9400 Å^22^) was used. The atom coordinates were optimized using the implemented routine in the FPLO software.

## 3 Results and Discussion

### 3.1 Crystal Structure

In this work, we revisit the crystal structure of the Fe–Ge NCL phase using synchrotron XRD data measured on a polycrystalline sample synthesized with nominal composition Fe_2_Ge_3_. The crystal structure of the Fe_2_Ge_3_ sample was refined as incommensurately modulated (Figure 1a), consistent with the FeGe_1.52_ structure reported by Li et al.^20^ The diffraction pattern contains both main reflections and incommensurate satellite reflections along the c* direction (Figure S2a,b). The presence of intense reflections at high 2θ angles indicates the formation of a composite, incommensurately modulated structure rather than a simple commensurate phase (Figure S2a,b). Phase analysis also revealed the presence of trace amounts of the FeGe phase. Experimental details and crystallographic data for the modulated FeGe_1.52_ structure are summarized in Table 1, and experimental, calculated, and difference between experimental and calculated X-ray powder diffraction patterns of the sample are shown in Figure 1. The composition FeGe_1.52_ gives rise to a special case for the modulation vectors of the two sublattices: values of the γ vectors for Fe- and Ge-subcells are identical and correspond to the ratio *c*_Fe_/*c*_Ge_. Atomic coordinates and positional modulation parameters for atoms are listed in Tables 2 and S1, respectively. The modulated crystal structure is completely described with only two atomic positions. The Fe substructure represents a diamond-type network with small atomic displacements from the average positions. It was described by a single atomic position with two positional modulation waves. To describe the atomic arrangement in the Ge substructure, in which Ge atoms form helices along the crystallographic direction [001] (*c* direction), a single modulated atomic position with pronounced deviations from its average position is enough. Six positional modulation waves were introduced to construct the helices. Notably, the refined crystal structure is essentially identical to that previously reported in ref 21.

**Figure 1:**
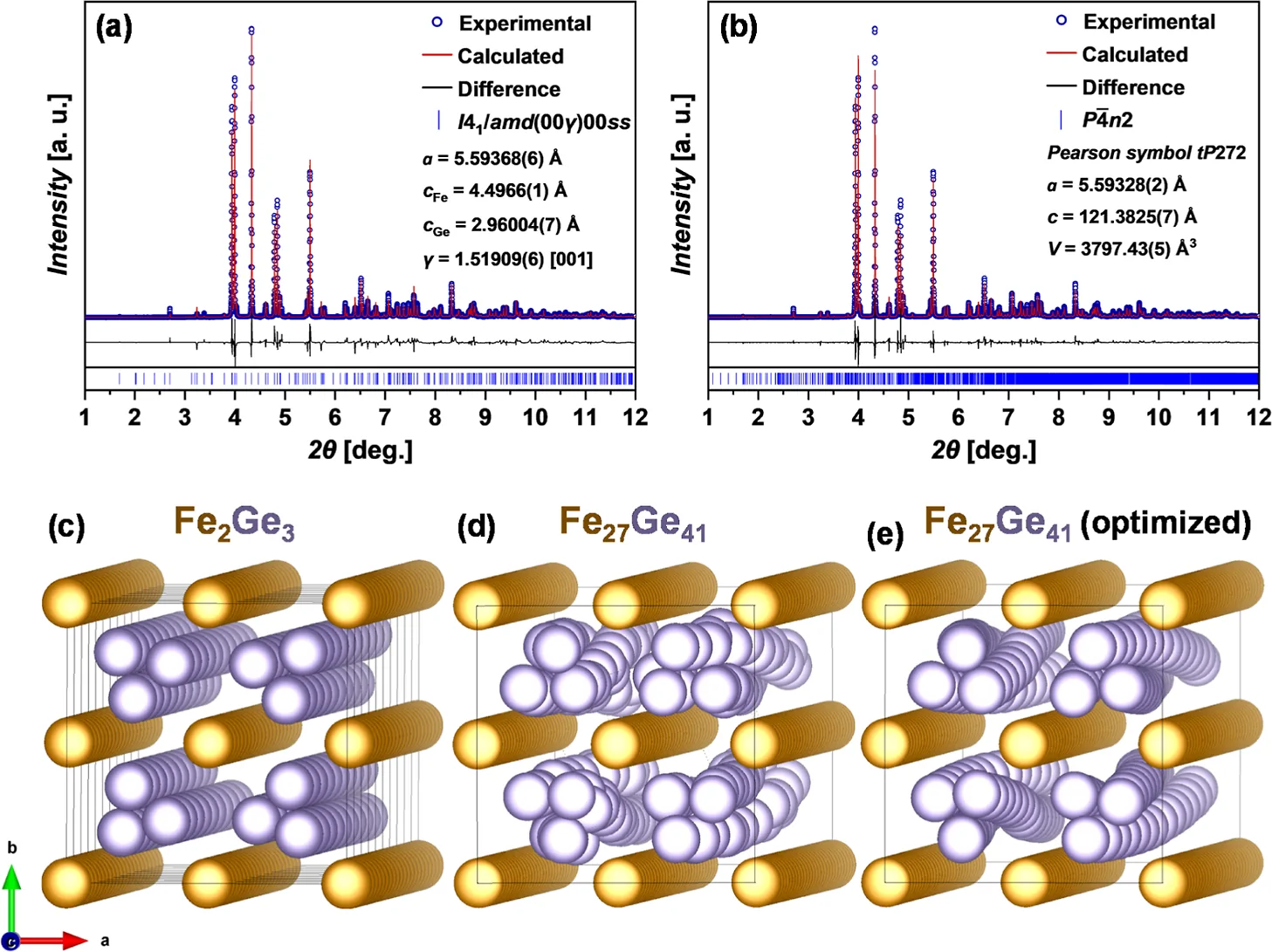
Powder synchrotron X-ray diffraction pattern of prepared Fe_2_Ge_3_ (nominal composition) sample with refinement results for (a) the incommensurately modulated structure in the superspace group *I*4_1_/*amd*(00γ)­00*ss* and (b) commensurate approximant structure in the space group *P*4̅*n*2. Crystal structure view along approximately [001] of (c) the Fe_2_Ge_3_,^22^ (d) Fe_27_Ge_41_ (refined), and (e) Fe_27_Ge_41_ (optimized).

**Table 2:** Atomic Coordinates and Isotropic Displacement Parameters for FeGe_1.52_

site	multiplicity	*x*	*y*	*z*	*B*_eq._ ^a^, Å^2^
Fe	4	0	1/4	3/8	1.25(2)
Ge	4	1/4	0	1/4	1.30(2)

a *B*_eq._ = 1/3­[*B*_11_ a*^2^ *a* ^2^ + ... 2*B*_23_ b*c*bc × cos α].

In the (3 + 1)­D superspace description, interatomic distances are functions of the superspace coordinate *x*_4_. The distribution of the interatomic distances in the FeGe_1.52_ structure as a function of the *x*_4_ coordinate is shown in Figure S2. The shortest homoatomic Fe–Fe and Ge–Ge interatomic distances cover ranges 2.99–3.05 Å and 3.05–3.25 Å, respectively, and are larger than the sums of corresponding atomic radii (*r*_at.(Fe)_ = 1.24 Å, *r*_at.(Ge)_ = 1.225 Å^44^). In contrast, the shortest heteroatomic Fe–Ge distances (2.20–2.70 Å) are compatible with the sums of atomic and covalent (*r*_cov.(Fe)_ = 1.165 Å, *r*_cov.(Ge)_ = 1.22 Å) radii, indicating strong heteroatomic interactions. Concluding from the analysis of the interatomic distances, the heteroatomic interactions seem to play a more important role compared with the homoatomic interactions. This is discussed further in Section 3.2.

Because standard solid-state DFT methods cannot treat aperiodic structures, we constructed a commensurate approximant of the FeGe_1.52_ structure for electronic structure calculations and property modeling. An approximant with the composition Fe_27_Ge_41_ was derived by applying the shifts from the ideal positions given by the modulation functions for the Fe and Ge atoms, yielding the structure shown in Figure 1d. Structural parameters were refined by the Rietveld method (Figure 1b and Table 1). Refined and standardized atomic coordinates and isotropic displacement parameters for the approximant Fe_27_Ge_41_ structure are given in Table S2. The unit cell of the Fe_27_Ge_41_ approximant contains 272 atoms that are distributed among 49 independent crystallographic positions: 28 sites are occupied by Fe atoms, and 21 sites are occupied by Ge atoms.

### 3.2 Chemical Bonding Analysis

The traditional interpretation of the NCL structures in general, and that of Fe_2_Ge_3_ in particular, in terms of a chimney–ladder construction (Figure 1c–e) involves homoatomic distances on the level of 2.99–3.05 Å for Fe–Fe and 3.05–3.25 Å for Ge–Ge contacts (Figures S1 and 2a). Moreover, consequent application of such a distance range for Ge–Ge results also in the interspiral connections (Figure 2a, green dashed line). Both distance groups are essentially larger than the sums of corresponding atomic or covalent radii. On the other hand, a variety of essentially shorter heteroatomic Fe–Ge distances in the range of 2.31–2.65 Å can be found in the commensurate structure, in the approximants of the incommensurate structure, and in the incommensurate structure itself, which raises the question about the bonding background of an interpretation.

**Figure 2:**
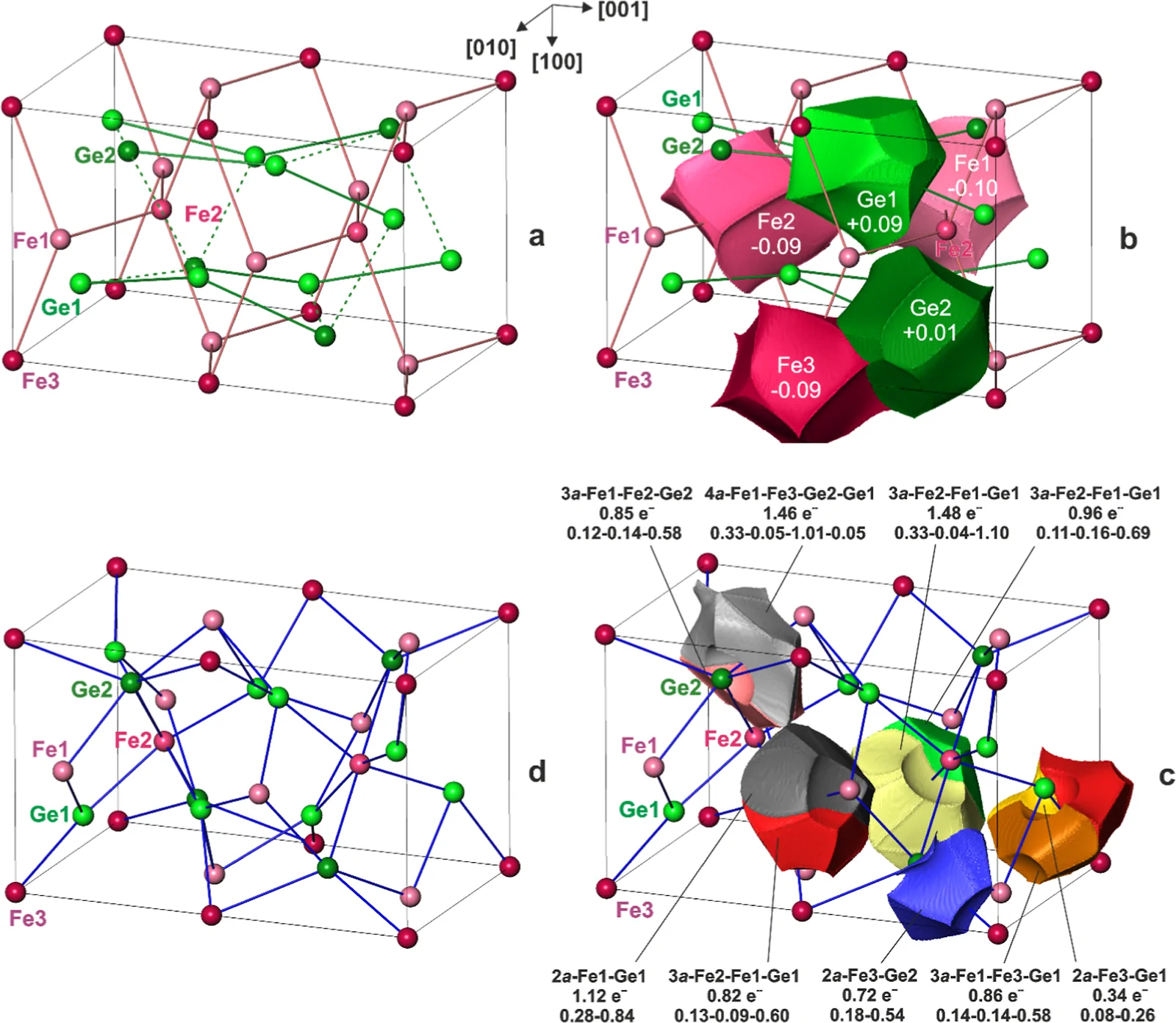
Crystal structure and chemical bonding in Fe_2_Ge_3_: (a) homoatomic contacts in the chimney (reddish) and ladder (green, solid for intrahelix, dashed for interhelix distances) “substructure”; (b) shapes and effective charges of atomic species after Bader^43^ obtained from the topological analysis of electron density; (c) ELI-D bond basins and their populations obtained from the combined ED/ELI-D analysis,^38^ for each basin are listed its atomicity (number of participating atoms, first line), electronic population (second line) and contributions of the participating atoms (in *e* ^–^, third line); (d) crystal structure as framework of heteroatomic contacts (blue), as revealed by the bonding analysis.

The effective charges of atomic species, obtained from the topological analysis of electron density (Figure 2b), reveal the unusually low charge transfer for intermetallic compounds: while the iron species carry a negative charge of −0.10 to −0.09, the germanium counterparts play the role of cations with charges of +0.09 and +0.01. This finding indicates that the bond energy in Fe_2_Ge_3_ is defined by the covalent contributions, with only a small part from the Coulombic interactions (cf.^45^ for calculation strategy).

Topological analysis of the distribution of the electron localizability indicator—a functional describing the behavior of the electron pairs—yields information about the atomic interactions—bonds—in position space. Intersection of the atomic basins with a bond basin of interest allows the detailed analysis of the atomic contribution to each bond. In Fe_2_Ge_3_ (Figure 2c), no homoatomic Ge–Ge and Fe–Fe bonds, which would be necessary as a bond backbone for the traditional “chimney–ladder” structure interpretation, were found. All bond basins are located in the vicinity of the heteroatomic connection lines (blue in Figure 2c). Therefore, from the bonding point of view, the crystal structure of Fe_2_Ge_3_ should be characterized as a three-dimensional framework of heteroatomic Fe–Ge bonds (Figure 2d). The familiar chimney–ladder construction should be viewed only as a crystallographic representation for visualizing the structure incommensurability.

With one exception, the bonds in Fe_2_Ge_3_ are either two- or three-atomic. The main contributions to each basin are made by the germanium species, i.e., all bonds are (moderately) polar. The variety of different bonds (especially considering the incommensurability of the crystal structure) is consistent with the low thermal conductivity measured recently on single crystals.^23^ Furthermore, there is no essential difference in the distribution of different bond types along different crystallographic directions. That is, the bonding can be described as inhomogeneous isotropic.^46^ Therefore, the lattice thermal conductivity should not show a strong anisotropy. The electronic contribution, on the other hand, will follow the electrical conductivity discussed below and thus is expected to show strong anisotropy with reduced value along the *c* direction.

### 3.3 Electronic Structure Calculations

Figure 3 shows results of the electronic structure for the optimized approximant Fe_27_Ge_41_ (Figure 1e). In Figure 3a, electronic total densities of states (DOS) are presented for Fe_27_Ge_41_ as well as for the optimized commensurate Fe_2_Ge_3_ model (atomic positions are listed in Table S3 in the Supporting Information). If not specified otherwise, we will refer to the calculations performed for the optimized structures. Electronic structure computation for the commensurate phase was initially performed with spin polarization enabled; however, it converged to a nonmagnetic state, in agreement with the nonmagnetic ground state of this system;^22^ thus further calculations were carried out without spin polarization. Starting with the case of Fe_2_Ge_3_, the electronic structure, i.e., the general shape of the DOS and the dispersion relation, is in good agreement with the literature,^22,25^ apart from the band gap width, which will be discussed later. However, calculations for the basic Fe_2_Ge_3_ are unable to explain the heavily *n*-type character of the system (*n* > 2 × 10^21^ cm^–3^), as the Fermi level is located at the edge of the valence bands. This is not the case for the Fe_27_Ge_41_ approximant, as here the ab initio calculations accurately predict the location of *E*_F_ in the conduction band (CB), which explains the *n*-type character with no need to look for a large amount of additional defects in terms of carrier concentration adjustment. The reason for this difference is the following. In NCL phases, as discussed already in literature,^13^ the gap opens at 14 valence electrons per transition metal (VEC = 14 per TM). Fe has 8 valence electrons, and Ge has 4; thus, in the case of Fe_2_Ge_3_, VEC equals to 28 per f.u. (14 per Fe), leading to the gap opening exactly above *E*_F_ when the Fe/Ge ratio is 1:1.5. In the large unit cell approximant, the stoichiometry is slightly different. Fe_27_Ge_41_ is approximately Fe_1_Ge_1.52_, making the system Ge-rich, and the additional electrons of Ge make the system *n*-type. Counting precisely the electrons per unit cell, there are 4 f.u. of Fe_27_Ge_41_ in the cell. The band gap opens at VEC = 14 per Fe atom; here at VEC = 4 × 27 × 14 = 1512. In the unit cell, however, there are 4 × (27 × 8 [Fe] + 41 × 4 [Ge]) valence electrons, which add up to 1520 (the unit cell volume is 3797 cm^3^). These additional 8 electrons per unit cell occupy the CB region above the gap, leading to a natural nominal *n*-type carrier concentration of *n*_theor_ = 8/(3797 cm^3^) = 2.1 × 10^21^ cm^–3^, which is very close to the one observed experimentally. Recalculating per Fe atom, the VEC is 14.074, explaining the *n*-type character according to the 14-VEC rule.

**Figure 3:**
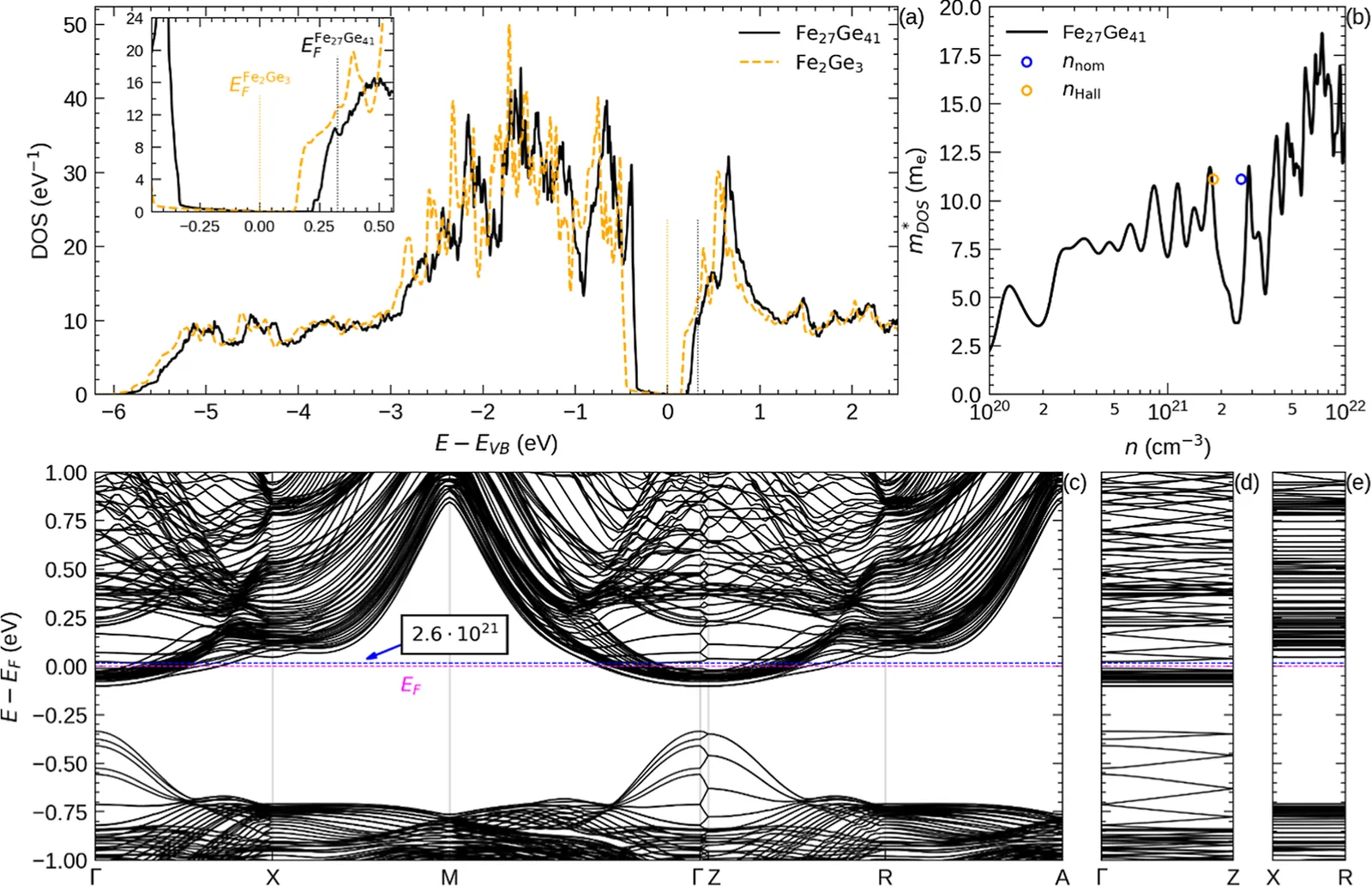
(a) Total density of states for Fe_27_Ge_41_ and Fe_2_Ge_3_ with the inset showing magnification in the energy range close to the band gap. Here, the energy scale is with respect to the valence band edge to allow easier comparison. The position of *E*_F_ in both structures is marked by lines. Total DOS of the Fe_27_Ge_41_ was divided by 13.5 to match the number of electrons in the valence band of the Fe_2_Ge_3_ unit cell. (b) Calculated density-of-states effective mass m*_DOS_. Experimental value of m*_DOS_ is marked at 2 positions, assuming the experimental Hall carrier concentration is equal to the nominal one, *n*_nom_ = 2.6 × 10^21^ cm^–3^, as well as using the value corrected by the Hall factor (see below) for *n*_Hall_ = 2.6 × 10^21^ cm^–3^ (which corresponds to *n*_nom_ = 1.89 × 10^21^ cm^–3^). (c–e) Electronic dispersion relation for Fe_27_Ge_41_. The horizontal dotted line represents the Fermi level position for *n*_nom_ = 2.6 × 10^21^ cm^–3^.

Thus, the proposed crystal structure model is able to explain the heavily *n*-type character of the material. A similar relationship between incommensurate structure and carrier concentration has been reported for MnSi_1.75–*x*_, where Si deficiency controls the electronic transport and leads to *p*-type behavior,^47^ in contrast to the Ge excess and *n*-type character observed in the present Fe–Ge system.

The overall shape of the total DOS at large scale is similar to that of Fe_2_Ge_3_, with some small differences visible, especially close to the band gap; as in the case of Fe_2_Ge_3_, the main part of the valence band is shifted toward lower energies. Partial DOS is shown in Figure S3, which indicates a high contribution of Fe 3d states in the vicinity of the Fermi level as well as at the edge of the valence band (VB), in contrast to the results available in the literature,^22^ where the top of the VB consisted mainly of Ge 4p states.

The electronic dispersion relation for Fe_27_Ge_41_ is shown in Figure 3c–e along high symmetry points. Performing a direct comparison with the band structure of commensurate Fe_2_Ge_3_ is challenging, as the electronic structure was calculated for a unit cell with 272 atoms, leading to a large number of bands. Despite that, the electronic dispersion relation of the Fe_27_Ge_41_ retains key features of the Fe_2_Ge_3_ (see Figure S4) calculated in this work, as well as when compared to the literature.^22,25^ Starting with the band gap, for Fe_27_Ge_41_, a direct band gap of 0.23 eV is observed at the Γ point, which is close to the value obtained in the case of the Fe_2_Ge_3_ commensurate structure (0.17 eV), as well as to the value estimated from the Goldsmid–Sharp formula of about 0.2 eV, as will be discussed later. It is worth mentioning, though, that in the case of Fe_2_Ge_3_, the calculations predict an indirect band gap, as the CB minimum is not located in any high-symmetry point, but along the Γ-*Z* direction. When it comes to comparison of the gap values with literature, our calculated results differ from the reported ones (0.11 eV,^22^ 0.2 eV^25^), which is primarily caused by the different exchange–correlation potential used in our case. The DFT calculations overall predict larger values than the estimation reported in ref 22 based on the high-temperature resistivity fit with the Arrhenius law (30 meV).

The bottom of the CB consists of multiple, low-dispersive bands located around the Γ point, and as many as 30 bands cross the Fermi level, which leads to a very high DOS effective mass. This is shown in Figure 3b, where m*_DOS_ (calculated with a method given in ref 48) is plotted as a function of carrier concentration. Near the Fermi level, a high m*_DOS_ of around 11 *m*_e_ (with *m*_e_ being the bare electron mass) is obtained. This result is in very good agreement with the experimental value of 11.1 *m*_e,_ even considering both nominal carrier concentration and carrier concentration corrected by the Hall factor. The average m*_DOS_ for a single band can be estimated to be ∼1 *m*_e,_ as 30 Γ-centered pockets contribute to the global m*_DOS_ and 11/30^2/3^ = 1.1. Additionally, we have analyzed the individual band masses using the *k*p method of ref 49 implemented in the mstar code. These calculations confirmed that the single-band effective masses are around 1 *m*_e,_ thus larger than in typical thermoelectric semiconductors. At the CB minimum at Γ, the *xx* (and *yy*) component of the effective mass tensor is 0.91 *m*_e_, while the *zz* component is 1.42 *m*_e,_ reflecting the anisotropy of the system with generally larger masses along the large *c* axis. For this band, the conductivity and DOS effective masses are equal to 1.04 *m*_e_ and 1.06 *m*_e_, respectively.

In contrast, the valence band edge has nearly a parabolic-like character with a small m*_DOS_ = 0.4 *m*_e_ and small DOS up to ∼−0.4 eV from the VB edge (Figure 3a). Additionally, when comparing the band structure of the large Fe_27_Ge_41_ approximant with Fe_2_Ge_3_, we observe that local minima of the bands in CB near the X and R points are no longer present, and in Fe_27_Ge_41_, bands along Z-R-A and Γ-X-M directions become very similar, which was not the case for the Fe_2_Ge_3_ unit cell. Also, the Fe_27_Ge_41_ approximant exhibits a flatter band structure in the *k*_z_ direction, which will affect the conductivity along the *c*-axis, discussed below.

### 3.4 Microstructure

Figure 4 presents the microstructural characterization of the sample with the nominal composition Fe_2_Ge_3_ after consolidating the powder using an SPS. The sample contains a noticeable amount of porosity, with a relative density of approximately 88% (Figure 4a,b). Attempts to obtain samples with higher density were unsuccessful due to the high hardness of the material and the limited sintering temperature, as further temperature increase leads to decomposition of the phase above 580 °C.^26^

**Figure 4:**
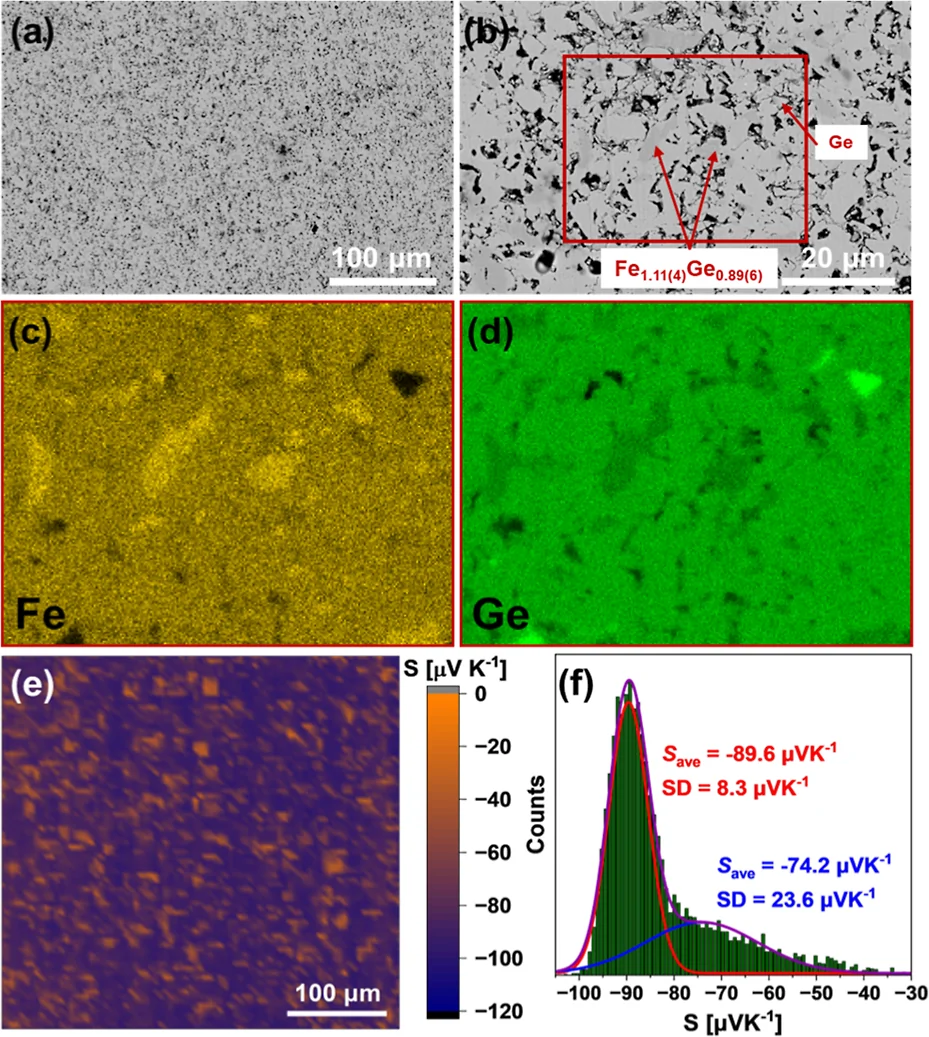
SEM backscattered images (a,b) and EDS elemental distribution maps (c,d) of the Fe_2_Ge_3_ sample. SThM map (e) and plotted histograms (f) of the Fe_2_Ge_3_ sample.

Since Fe_2_Ge_3_ forms via a peritectoid reaction between FeGe and FeGe_2_ at 580 °C,^26^ achieving a single-phase material is extremely challenging due to the slow kinetics of this transformation. Panels (b–d) reveal minor inclusions of the FeGe phase, which remained unreacted, as well as traces of Ge-rich microinclusions. This observation is consistent with the reported phase diagram, which indicates that FeGe_2_ becomes unstable below 530 °C.^26^ The presence of FeGe inclusions may also be attributed to the actual composition of the NCL phase, which is slightly richer in Ge (approximately FeGe_1.52_) rather than the nominal Fe_2_Ge_3_ stoichiometry.^20^

Consistent with the SEM observations, the Seebeck map reveals regions with lower absolute values of the Seebeck coefficient (cf. the SThM map in Figure 4e,f), which can be attributed to the presence of secondary phase inclusions and pores. The apparent size of these regions is somewhat larger, likely because of the limited spatial resolution of the SThM technique. The reduced Seebeck coefficient values observed in the inclusions suggest a higher carrier concentration in these regions, which consequently lowers the overall absolute Seebeck coefficient of the bulk sample.

### 3.5 Thermoelectric Properties

Table 3 summarizes the key thermoelectric parameters for the Fe_2_Ge_3_ polycrystalline sample at *T* = 300 K. It should be noted that this sample corresponds to the incommensurately modulated structure FeGe_1.52_ discussed above, and that all reported transport properties were measured on this same specimen. The reported quantities include the Seebeck coefficient *S*, electrical conductivity σ, thermal conductivity κ, Hall carrier concentration *n*_H_, Hall carrier mobility μ_H_, and the density-of-states (DOS) effective mass m*.

**Table 3:** Seebeck Coefficient *S*, Electrical Conductivity σ, Thermal Conductivity κ, Hall Carrier Concentration *n*_H,_ Hall Carrier Mobility μ_H_, and DOS Effective Mass m* for the Fe_2_Ge_3_ Samples at *T* = 300 K

compound	*S* [μV K^–1^]	σ [S cm^–1^]	κ [W m^–1^ K^–1^]	*n*_H_ [cm^–3^]	μ_H_ [cm^2^ V^–1^ s^–1^]	m*/me
Fe_2_Ge_3_	–95.7	942.4	2.17	2.6 × 10^21^	2.3	11.1
Fe_2_Ge_3_ single crystal (*c*-axis)^23^	–141	15	1.9	2.5 × 10^21^	0.037	-

For the investigated Fe_2_Ge_3_ polycrystalline sample, the Seebeck coefficient is approximately −100 μV K^–1^ at 300 K, indicating *n*-type conduction. The compound shows a relatively high electrical conductivity of ∼940 S cm^–1^, combined with a low thermal conductivity of 2.2 W m^–1^ K^–1^, which is favorable for thermoelectric applications. Consistent with already discussed DFT calculations, the Hall carrier concentration reaches 2.6 × 10^21^ cm^–3^, indicating an unusually high carrier density. Consequently, the Hall mobility remains relatively small (2.3 cm^2^ V^–1^ s^–1^), which comes from the flat band structure in the CB and the resulting high m*. In comparison, the Fe_2_Ge_3_ single crystal measured along the *c*-axis by Xu et al.^23^ exhibits a higher absolute Seebeck coefficient but dramatically lower electrical conductivity and Hall mobility (see Table 3), despite a comparable carrier concentration, highlighting the strong anisotropy and the substantial impact of microstructure on charge transport relative to our polycrystalline sample.

To gain deeper insight into the electronic transport properties, we calculated the DOS effective mass using the experimentally measured Seebeck coefficient and carrier concentration, assuming scattering at the deformation potential of the acoustic phonons as the main scattering mechanism (*r* = 0) and a reported bandgap value of *E*_g_ = 0.2 eV, as described in the Supporting Information. The calculations reveal that the DOS effective mass of Fe_2_Ge_3_ is approximately 11.1 *m*_e_ (Table 3). Such an unusually large value is noteworthy, as it indicates a substantial deviation from free-electron-like behavior. This enhanced DOS effective mass originates from the particular features of the band structure, discussed above, such as multiple flat bands near the Fermi level (Figure 3c). The result suggests that the electronic transport in Fe_2_Ge_3_ is governed not only by the high carrier concentration but also by the complex interplay between band topology and electron–electron interactions, which could have an important effect on its thermoelectric performance.

Figure 5 shows the temperature-dependent thermoelectric properties of Fe_2_Ge_3_ obtained in this study, along with previously reported data from Sato et al.^25^ for comparison. The Seebeck coefficient *S* remains negative across the entire temperature range, confirming *n*-type conduction (Figure 5a). At room temperature, *S* is about −100 μV K^–1^ and its magnitude increases with temperature, reaching roughly −160 μV K^–1^ at 600 K. Above this point, *S*(*T*) begins to decrease, most likely due to the activation of intrinsic carriers. Consistent with the carrier concentration dependence of the Seebeck coefficient, the electrical conductivity σ decreases from ∼950 S cm^–1^ at room temperature to ∼500 S cm^–1^ at 600 K, before showing an upturn (Figure 5b). A Goldsmid–Sharp estimate of the band gap (*E*_g_ = 2*S*_max_ *T*_max_) gives a value of ∼0.2 eV, which is close to the band structure result. However, this narrow band gap alone does not explain the onset of minority-carrier activation observed in both *S*(*T*) and σ­(*T*) in view of the transport properties calculations, discussed below.

**Figure 5:**
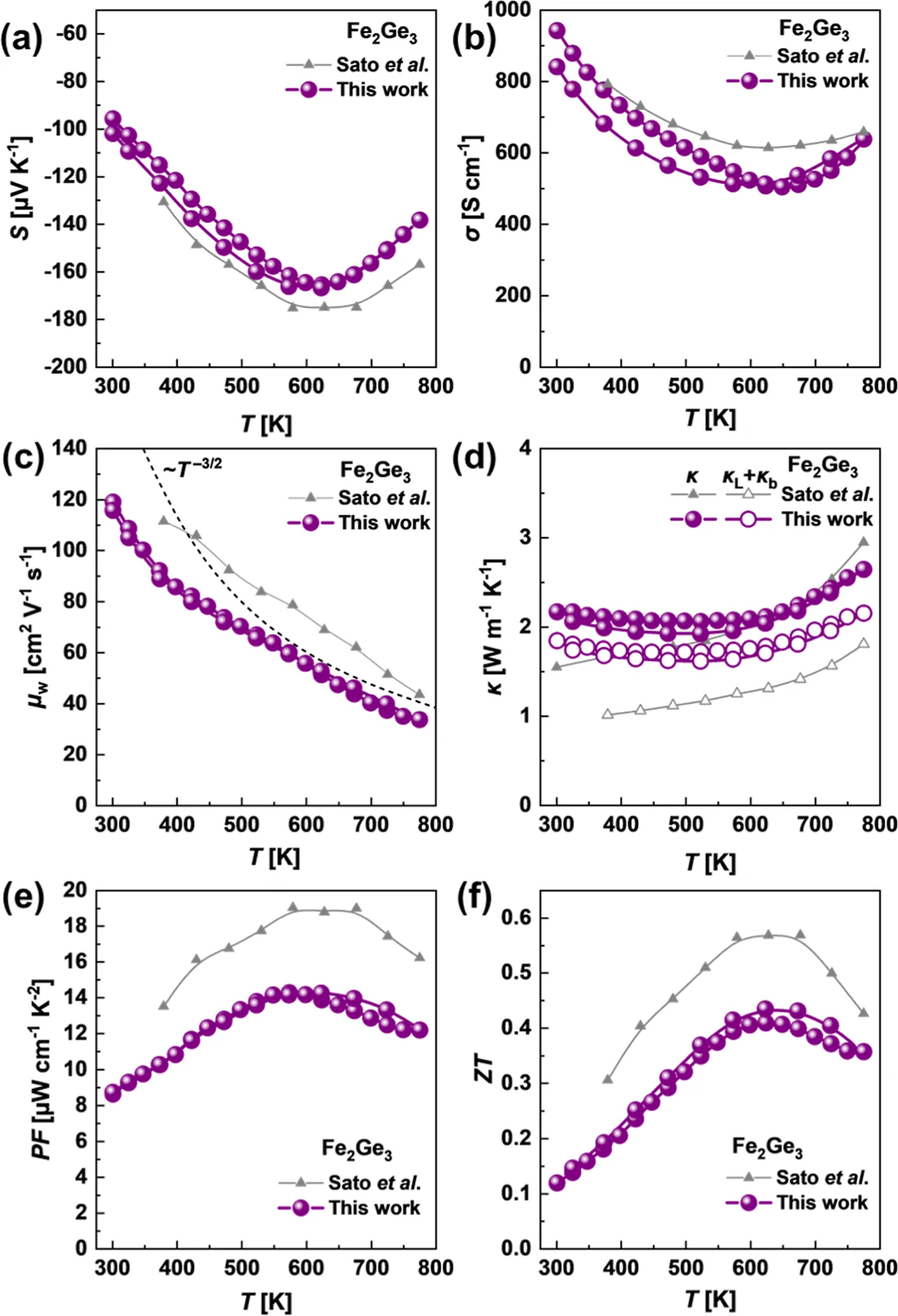
Temperature-dependent (a) Seebeck coefficient, (b) electrical conductivity, (c) weighted mobility, (d) total and lattice thermal conductivities, (e) thermoelectric power factor PF, and (f) figure of merit ZT of the Fe_2_Ge_3_ polycrystalline samples, measured during both heating and cooling cycles. Data reported by Sato et al.^25^ were used for comparison.

Transport properties for the approximant Fe_27_Ge_41_ were calculated using the Boltzmann theory within the constant relaxation time approximation (CRTA). As a consequence, electrical conductivity σ and power factor PF depend linearly on the relaxation time, making the Seebeck coefficient the only quantity possible to directly compare with the experiment. In these transport computations, the bandgap value obtained from the electronic structure calculations (0.23 eV) was used, unless specified otherwise. In Figure 6a, the Pisarenko plot is shown with *n*-type effective (polycrystalline) thermopower calculated using the parallel grain model^48^ for selected temperatures. In addition, diagonal elements of the transport properties tensor are also shown: in the in-plane (*a*) and along the *c*-axis (*c*) due to the tetragonal crystal structure of the system. The thermopower of Fe_27_Ge_41_ exhibits a large degree of anisotropy with the *S*_c_ dominating in vicinity of the experimental carrier concentration (∼2 × 10^21^ cm^–3^), although the dominant direction reverses near to the peak *S* value for 300 and 700 K. Generally, decreasing the carrier concentration could vastly increase the Seebeck coefficient, e.g., close to the 700 K, the calculations predict over 300 μV/K near 3 × 10^20^ cm^–3^ compared to a maximum of ∼170 μV/K considering nominal *n* = 2.6 × 10^21^ cm^–3^. Unfortunately, the benefit from such a change in terms of thermoelectric conversion efficiency is restricted as the optimal *n* from the perspective of the PF is in the range of 2–3 × 10^21^ cm^–3^ (Figure 6b), which is close to the *n* of our samples. This behavior is caused by constantly decreasing electrical conductivity when moving *E*_F_ closer to the CB edge (Figure S5) and indicates limited potential in terms of PF enhancement through further optimization of carrier concentration.

**Figure 6:**
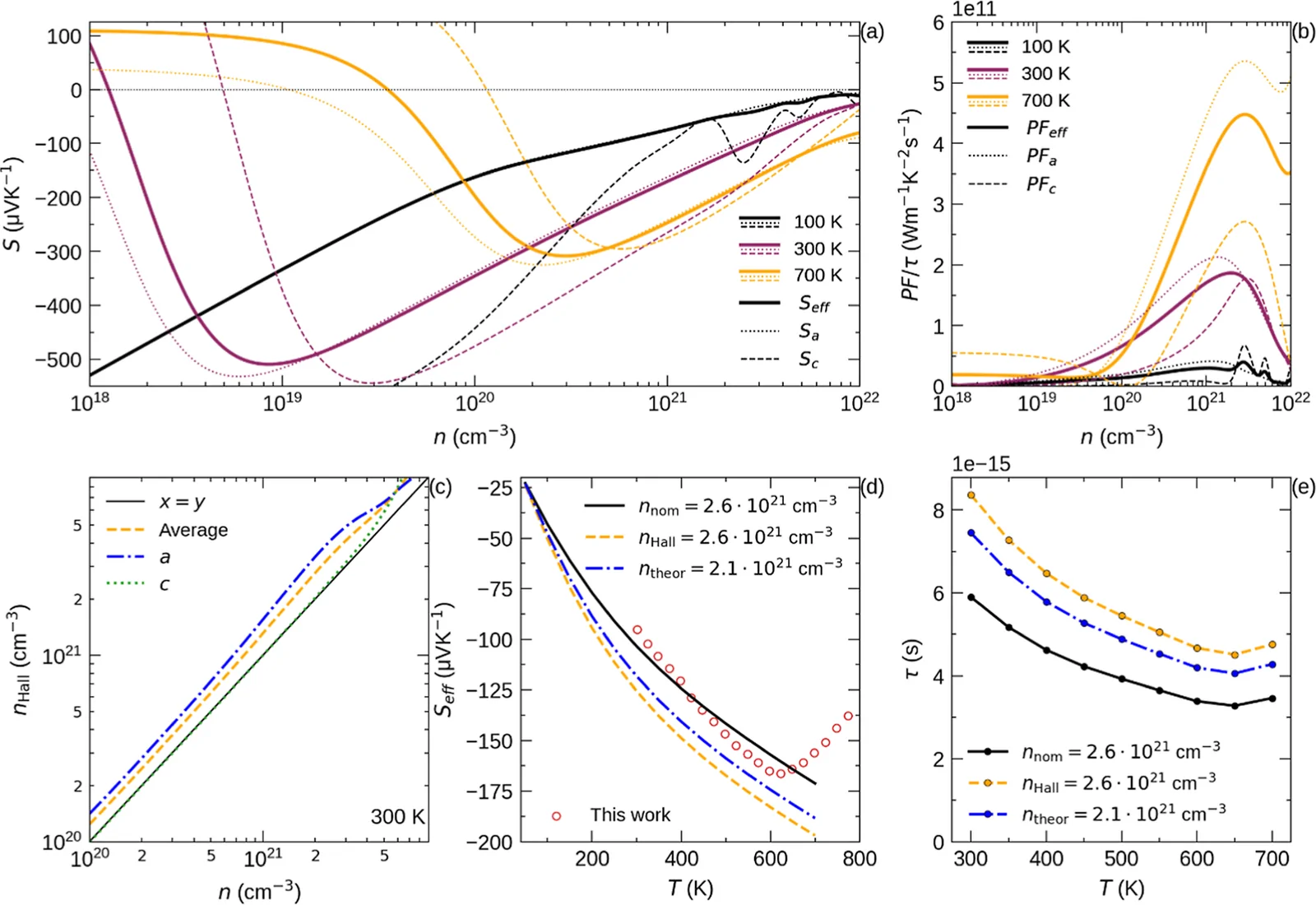
Calculated transport properties for the approximant Fe_27_Ge_41_. (a) Calculated *n*-type Seebeck coefficient at *T* = 100, 300, and 700 K as a function of carrier concentration. (b) Calculated power factor divided by the relaxation time τ as a function of carrier concentration for selected temperatures. (c) Hall carrier concentration *n*_Hall_ = 1/*eR*_H_ as a function of nominal carrier concentration *n* for 300 K. (d) Temperature-dependent effective thermopower and (e) relaxation time τ assuming 3 values of carrier concentrations: *n*_nom_ = 2.6 × 10^21^ cm^–3^, carrier concentration which results in the Hall carrier concentration of *n*_Hall_ = 2.6 × 10^21^ cm^–3^ as well as assuming the theoretical value for the ideal stoichiometric Fe_27_Ge_41_ of *n*_theor_ = 2.1 × 10^21^ cm^–3^.

The σ/τ itself also displays a high degree of anisotropy, as shown in Figure 7a, where magnification of electrical conductivity close to the CB edge is plotted. In the case of Fe_27_Ge_41_, the *c*-direction electrical conductivity σ_*c*_ is noticeably lower in comparison to the in-plane σ_*a*_ or average conductivity, denoted as σ_tr_. This difference is shown in Figure 7b, where the ratio of σ_*c*_/σ_*a*_ and σ_*c*_/σ_tr_ is presented, and here, the Fe_27_Ge_41_ displays higher anisotropy in this energy region compared to predictions based on the smaller Fe_2_Ge_3_ unit cell model. For the location of *E*_F,_ which corresponds to the Hall carrier concentration of *n*_Hall_ = 2.6 × 10^21^ cm^–3^, the values of both σ_*c*_/σ_*a*_ and σ_*c*_/σ_tr_ are close to 0.1 in Fe_27_Ge_41_. The leading cause of such large anisotropy is the presence of almost dispersionless bands along the *k*_z_ direction at the bottom of CB (Figure 3c–e) in the Fe_27_Ge_41_ approximant. Low values of σ_*c*_ in Fe_27_Ge_41_ also partially explain the very low conductivity and Hall carrier mobility that was reported in ref 23 (measured in a single crystal along the *c*-axis) in comparison to our samples, while the carrier concentration in both cases remained similar (see Table 3). This anisotropy is most likely enhanced by differences in electronic relaxation times between the in-plane and along the *c*-axis directions.

**Figure 7:**
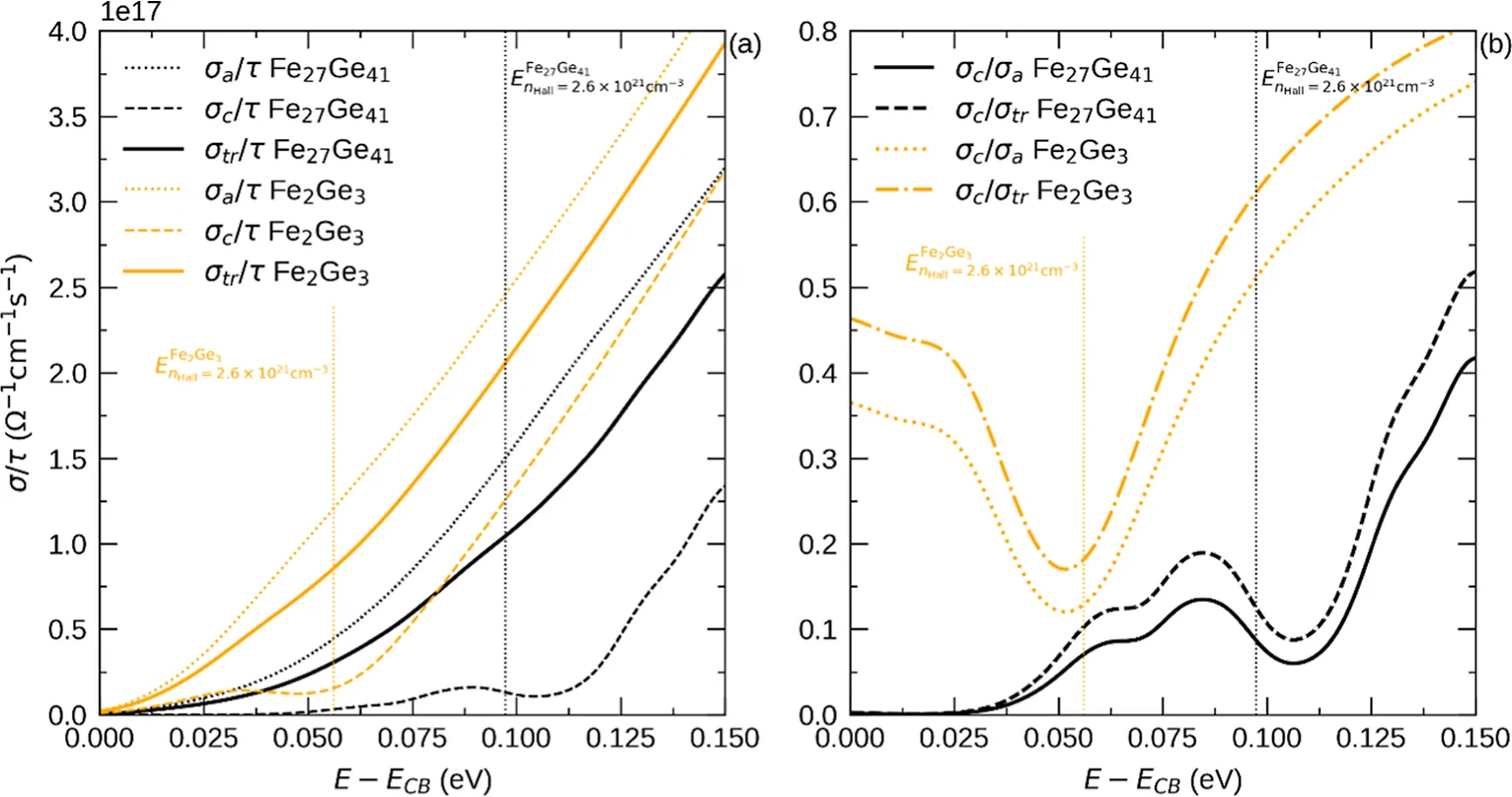
(a) Magnification of energy-dependent electrical conductivity σ over relaxation time τ close to the conduction band edge for Fe_27_Ge_41_ and Fe_2_Ge_3_ at 50 K as a function of the chemical potential μ (b) ratio of electrical conductivity along the *c* direction σ_*c*_ to the in-plane σ_*a*_ as well as average conductivity σ_tr_ for Fe_27_Ge_41_ and Fe_2_Ge_3_ as a function of the chemical potential μ. Vertical lines correspond to the position of the Fermi level where carrier concentration is equal to *n*_Hall_ = 2.6 × 10^21^ cm^–3^.

To facilitate an accurate comparison of calculated *S* with the experimental measurements, elements of the Hall coefficient tensor *R*_H_ were analyzed, as the carrier concentration measured through Hall effect measurements generally deviates from the actual nominal carrier concentration due to anisotropy of the band structure. This is shown in Figure 6c, where Hall carrier concentration from the usual free-electron-like formula, *n*_H_ = 1/*eR*_H_, is plotted as a function of nominal carrier concentration *n* at 300 K. In the tetragonal crystal structure, two independent components of *R*_H_ exist, that is, for the magnetic field in the in-plane direction (denoted as *a*) and along the *c* axis (denoted as *c*). Close to the values of experimental *n*, small anisotropy is observed for the *a* component, as well as considering the average of the independent components, meaning that a correction, Hall factor *r*_H_, should be used: *n*_nominal_ = *r*_H_ *n*_H_ = *r*_H_/*eR*_H_. Here, we assume that the measured Hall resistivity corresponds to the trace of the *R*_H_ tensor, in which *r*_H_ is 0.726. This means that the experimental measurement overestimates the nominal values by nearly 38% without the Hall factor applied, and in further discussion, we will refer to the corrected experimental *n* as *n*_Hall_, which corresponds to the nominal concentration of 1.89 × 10^21^ cm^–3^.

In Figure 6d, to compare the calculations of the temperature-dependent effective thermopower *S*_eff_ (*S*_eff_ is the weighted-average thermopower with conductivity in each direction as a weight) with experiments, three theoretical curves are plotted, corresponding to three carrier concentrations: nominal *n*_nom_ = 2.6 × 10^21^ cm^–3^, corrected via Hall factor for *n*_Hall_ = 2.6 × 10^21^ cm^–3^, as well as assuming the theoretical value of *n* considering the number of valence electrons in the stoichiometric unit cell of Fe_27_Ge_41,_ *n*_theor_ = 2.1 × 10^21^ cm^–3^. Theoretical results are in good agreement with the experiment, considering the nature of CRTA, as, in the worst case, our calculations overestimate measured values by about 20–30 μV/K.

What is not reproduced in calculations for any carrier concentrations included in Figure 6d is the onset of the bipolar conduction effect, which is seen in the measurement at around 600 K as the decreasing thermopower (in absolute value). This discrepancy is observed even though the theoretical bandgap used in the calculations is near the Goldsmid–Sharp estimation, based on the experiment. This is a consequence of the strong asymmetry in conductivities of the conduction and valence bands, where the much larger σ­(*E*) in the latter (see Figure S5) effectively weakens the bipolar effect. It is also worth noting that even with a strongly reduced gap size in calculations, we have not observed an onset of the bipolar effect, as shown in Figure S6. That suggests that the electron–phonon relaxation time, not included in our CRTA calculations, strongly reduces the asymmetry between the conductivities in conduction and valence bands, compared to the CRTA prediction. Another possibility is the existence of small amount of defects in the structure that would not alter the carrier concentration meaningfully might also be detrimental to thermopower and intensify the bipolar effect, as observed, e.g., in SnBi_2_Te_4_.^50^ However, analysis of the relaxation time obtained here using the calculated and measured electrical conductivity yields τ in the range of 6–8 fs at 300 K, suggesting that the concentration of defects in samples is not overwhelming. Nevertheless, the agreement of the calculated thermopower with experiment is quite satisfactory up to 600 K and indicates the correctness of the calculated band structure, but the onset of bipolar effects must be further clarified.

Returning to the experimental results, the carrier-independent weighted mobility decreases across the entire temperature range and follows an approximate *T* ^–3/2^ dependence (Figure 5c), which is commonly associated with phonon-limited charge transport in thermoelectric semiconductors. We note, however, that mobility temperature dependences alone do not uniquely identify the dominant scattering mechanism in complex materials.^51^ A slight deviation from the *T* ^–3/2^ trend at lower temperatures may be attributed to additional grain boundary scattering.

The total thermal conductivity κ, presented in Figure 5d, decreases slightly from 2.1 W·m^–1^·K^–1^ at 300 K to 2.0 W·m^–1^·K^–1^ at 550 K, before rising at higher temperatures. This increase is indicative of the onset of bipolar conduction, where thermally excited minority carriers contribute to heat transport.

The energy conversion performance of the investigated Fe_2_Ge_3_ sample was evaluated by calculating the power factor (PF = *S* ^2^σ) and the dimensionless thermoelectric figure of merit ZT (Figure 5e,f). The power factor reaches nearly 9 μW·m^–1^·K^–2^ at 300 K and attains a maximum of 14 μW·m^–1^·K^–2^ around 600 K. The ZT also reaches a promising value of 0.45 at 600 K. For comparison, the values reported by Sato et al.^25^ (ZT = 0.57) are somewhat higher, mainly due to their lower lattice thermal conductivity and slightly larger PF. The lower PF in our samples can be attributed to reduced carrier mobility caused by enhanced grain-boundary scattering, while the higher κ_L_ may arise from differences in the defect subsystem. It should be noted that direct comparison with literature data of Sato et al.^25^ should be treated with caution due to differences in starting composition, modulation parameter γ, phase purity, and synthesis conditions. In particular, Sato et al. reported a minor FeGe_2_ secondary phase, likely related to the slightly Ge-rich starting composition (Fe/Ge = 39:61), whereas in the present work, FeGe is observed, consistent with the nominal composition (Fe/Ge = 40:60) and the Fe–Ge phase diagram, given that the FeGe_1.52_ NCL phase corresponds to Fe/Ge ≈ 39.2:60.8. Nevertheless, the consistently low lattice thermal conductivity and comparable thermoelectric performance reported across studies suggest that these properties are intrinsic to the incommensurate Fe–Ge phase. Despite these differences, the measured thermoelectric performance clearly demonstrates the high potential of Fe_2_Ge_3_ for further optimization and improvement of its thermoelectric properties. According to our DFT results, the calculated CRTA power factor, shown in Figure 6b, shows that the optimal carrier concentration is between 2 and 3 × 10^21^ cm^–3^, which is in the range observed in the studied samples and limits the expected improvement due to changes in carrier concentration. The larger PF values may, however, be obtained for the preferentially ordered samples or single crystals in the *ab* in-plane direction, as the transport along the *c*-axis is hampered due to the low electrical conductivity in that direction. Also, band engineering effects related to doping may be further explored to possibly improve the power factor.

### 3.6 Thermal Transport Properties

To gain insight into the mechanisms of thermal transport in the investigated material, we estimated the lattice thermal conductivity, κ_L_, using the relation κ_L_ = κ – κ_e_ – κ_b,_ where κ_e_ and κ_b_ are the electronic and bipolar contributions to the total thermal conductivity. The electronic component κ_e_ was calculated using the Wiedemann–Franz law (κ_e_/σ = *LT*, where *L* is the Lorenz number). The Lorenz number was determined using the approximation procedure proposed by Kim et al.^52^ and the Kane band model,^53,54^ as described in the Supporting Information. Notably, the Lorenz number obtained by these two approaches differs quite significantly: 1.9 × 10^–8^ WΩK^–2^ and 1.3 × 10^–8^ WΩK^–2^ at 300 K for the first and second methods, respectively (Figure S8a). This discrepancy can be attributed to the different assumptions regarding band nonparabolicity and carrier scattering mechanisms inherent to each model. The Lorenz number obtained from the Kane band model was used to calculate the κ_L_ (Figure 5d).

The lattice thermal conductivity of the investigated material is very low, with a value of approximately 1.85 W·m^–1^·K^–1^ at room temperature, decreasing to about 1.7 W·m^–1^·K^–1^ at 550 K (Figure 5d). Up to 550 K, the bipolar term can be neglected, allowing us to consider only the lattice thermal conductivity values. Such low values of κ_L_ are comparable to, or even lower than, those observed in classic narrow-bandgap thermoelectric chalcogenides such as Bi_2_Te_3_,^55^ PbTe,^56^ and GeTe.^3^ Previous first-principles studies have predicted anisotropic lattice thermal conductivity in Fe_2_Ge_3_ arising from direction-dependent optical-acoustic phonon hybridization, which enhances phonon scattering and suppresses heat transport along specific crystallographic directions.^57^

To investigate the origins of the unusually low lattice thermal conductivity in the investigated incommensurate NCL phase, we performed pulse-echo as well as resonant ultrasonic measurements and combined the obtained results with the Debye–Callaway approach calculations. Table 4 summarizes the elastic and thermal transport properties of the Fe_2_Ge_3_ polycrystalline sample.

**Table 4:** Elastic and Thermal Transport Properties of the Fe_2_Ge_3_ Polycrystalline Sample

method	*v*_l_, m s^–1^	*v*_t_, m s^–1^	*v*_m_, m s^–1^	Θ_D_, K	ν	γ	*B*, GPa	*E*, GPa	*l*ph, Å	κ_L_, Wm^–1^ K^–1^	κ_glass_, Wm^–1^ K^–1^
pulse echo	4644	2799	3095	367	0.21	1.34	87	149	8.33	1.85	0.98
RUS	4810	2790	3090	370	0.24	1.48	91	138	8.35	1.85	1.0

The longitudinal, transverse, and mean speed of sounds (*v*_l_, *v*_t_, and *v*_m_) are relatively high, at ∼4800, ∼2800, and ∼3100 m·s^–1^, respectively, arising from the high values of the bulk modulus (*B* = 87 GPa) and Young’s modulus (*E* = 149 GPa). The elastic moduli of Fe_2_Ge_3_ are comparable to SiGe and half-Heuslers, which are much stiffer than other thermoelectric materials.^3^ Further, the Debye temperature (Θ_D_ = 367 K), Poisson’s ratio (ν = 0.21), and Grüneisen parameter (γ = 1.34 from pulse echo and 1.48 from RUS) suggest only moderate lattice anharmonicity. The variation of the elastic constants with temperature, shown in Figure 8a, exhibits a gradual decrease with increasing temperature, indicating thermal softening. In Figure 8b, corresponding longitudinal (*v*_l_) and transverse (*v*_t_) sound velocities as a function of temperature are plotted. The room temperature results from the pulse echo method are also shown for comparison (triangular symbols). The mild decrease in the Young’s modulus (∼3% softening over the 400 K measurement range) further supports the finding that this compound is not highly anharmonic.^3^

**Figure 8:**
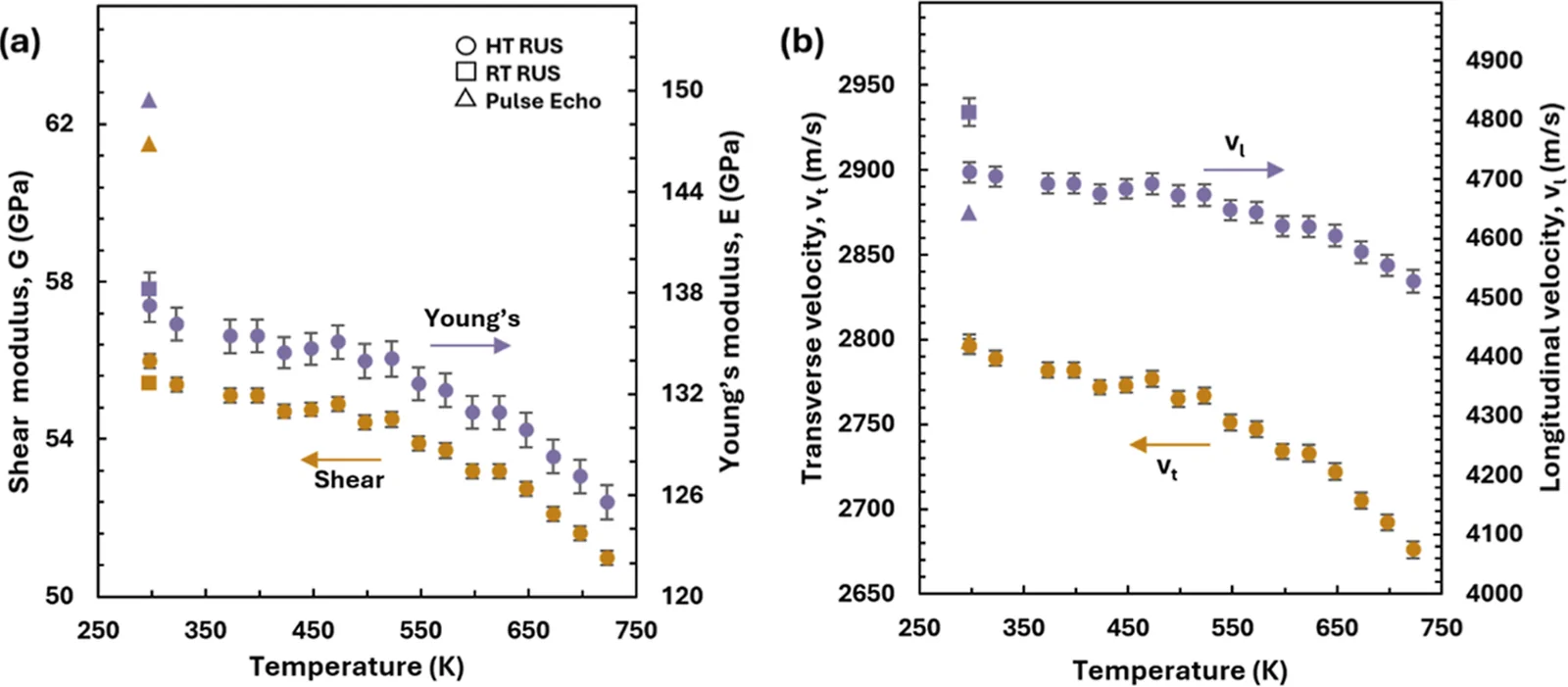
Temperature dependence of elastic constants and sound velocities.

Given the high mechanical stiffness and relatively harmonic bonding of Fe_2_Ge_3_, the low measured lattice thermal conductivity of 1.85 Wm^–1^ K^–1^ is surprising. Attempts to reproduce the lattice thermal conductivity using the Debye–Callaway model, accounting for Umklapp (U) and grain-boundary scattering, were unsuccessful, even when accurate temperature-dependent sound-velocity data were employed (Figure S8b). Point defects, which typically play a dominant role in suppressing phonon transport, were not detected by XRD analysis. Although XRD analysis alone does not provide sufficient sensitivity to reliably detect or exclude point defects, the combined structural and electronic structure analyses suggest that a large concentration of defects is unlikely. Therefore, point-defect scattering alone is not expected to account for the large discrepancy between the experimental and calculated lattice thermal conductivity values. Debye–Callaway modeling that includes only Umklapp and grain-boundary scattering (i.e., excluding point-defect scattering) predicts a room-temperature lattice thermal conductivity of approximately 13 Wm^–1^ K^–1^ (Figure S8b), which is significantly higher than the experimental value. In the Debye–Callaway calculations, a characteristic grain size of 5 μm was used, corresponding to the experimentally observed microstructure. Variations of the grain size within the micrometer range only weakly affected the calculated lattice thermal conductivity. Consequently, the unusually low κ_L_ of Fe_2_Ge_3_ corresponds to a short estimated phonon mean free path (*l*_ph_ ≈ 8.3 Å), which may be related to additional scattering associated with the incommensurate crystal structure and bonding inhomogeneity. It should be noted that the measured sample exhibits ∼12% porosity, which may contribute to additional phonon scattering and a reduction of the lattice thermal conductivity. However, similar low values of lattice thermal conductivity have been reported for fully dense single-crystalline Fe_2_Ge_3_,^22,23^ suggesting that porosity is not the sole origin of the low κ_L_ and that the structural complexity of the NCL framework may also contribute. Nevertheless, without direct phonon calculations, the relative contributions of these scattering mechanisms cannot be determined conclusively.

## 4 Conclusions

In this work, the structure–property relations for the Fe–Ge NCL phase near the nominal Fe_2_Ge_3_ composition are established. Combining high-resolution synchrotron XRD, thermoelectric and Hall measurements, elastic and ultrasonic characterization, first-principles calculations, and quantum mechanical position-space analysis of chemical bonding, the coexistence of degenerate *n*-type electronic transport and phonon-glass lattice thermal conductivity is shown to be an intrinsic consequence of slight stoichiometric deviation accommodated by an incommensurate NCL framework.

In agreement with the literature information, samples prepared with a nominal Fe_2_Ge_3_ composition crystallize as an incommensurately modulated NCL phase with a composition close to FeGe_1.52_, represented by a commensurate approximant, Fe_27_Ge_41_ (*tP*272), containing 272 atoms per unit cell. This approximant faithfully captures the essential structural motifs of the incommensurate phase while enabling direct first-principles electronic structure and transport calculations, which are not accessible within higher-dimensional crystallographic descriptions.

Real-space chemical-bonding analysis based on electron density and electron localizability indicator provides direct evidence that the NCL phase lacks homoatomic Fe–Fe or Ge–Ge bonds. Instead, the structure is formed by a three-dimensional framework of heteroatomic multiatomic Fe–Ge bonds with pronounced bonding inhomogeneity. This result establishes a direct link between structural complexity and local bonding heterogeneity.

Electronic-structure calculations based on the Fe_27_Ge_41_ approximant reveal that the slight Ge excess introduces eight additional valence electrons per unit cell, shifting the Fermi level into the CB. This stoichiometry-driven mechanism yields a nominal carrier concentration of 2.1 × 10^21^ cm^–3^, in good agreement with Hall measurements (*n*_H_ ≈ 2.6 × 10^21^ cm^–3^ at 300 K). The degenerate *n*-type behavior is, therefore, explained without invoking extrinsic donor defects or uncontrolled disorder. The calculated electronic structure shows a large DOS effective mass (∼11 *m*_e_) due to convergence of CBs near the Fermi level, with the resulting thermopower closely matching experimental measurements and supporting the proposed structural model.

The low lattice thermal conductivity of approximately 1.8 W m^–1^ K^–1^ at 300 K is established experimentally from direct measurements of the total thermal conductivity combined with an estimation of the electronic contribution using the Kane band model and is in agreement with pronounced bonding inhomogeneity. Estimates based solely on elastic properties and sound velocities substantially exceed the measured total thermal conductivity, indicating that elastic stiffness alone does not govern heat transport in the Fe–Ge NCL phase. The results indicate additional phonon scattering that limits the phonon mean free path to near-interatomic length scales and may be associated with the incommensurate structural complexity and bonding inhomogeneity.

## Supplementary Material



## Notes

Results of the X-ray diffraction analyses; results of the DFT calculations; calculated Lorenz number and lattice thermal conductivity; details of the Kane band model calculations; details of weighted mobility calculations; details of elastic property calculations; and details of the thermal transport property calculations (PDF)
