# Supplementary material for: Structure–Thermoelectric Property Relations of the Nowotny Chimney–Ladder Phase Fe2Ge3
Source: Chem Mater. 2026 Aug 17;38(19):9802–16. doi: 10.1021/acs.chemmater.6c00878 (PMC13673942; doi:10.1021/acs.chemmater.6c00878)
Supplement: Supplementary file 1 [file cm6c00878_si_001.pdf]

Supporting Information

Structure–Thermoelectric Property Relations of the Nowotny Chimney–Ladder Phase Fe_2Ge_3

Oleksandr Cherniushok[1], Illia Lukashchuk[1], Kacper Pryga[2], Raisa Tahiyah[3], Taras Parashchuk[1], Yaroslav Tokaychuk[4], Lev Akselrud[4], Bartlomiej Wiendlocha[2], Roman Gladyshevskii[4], Yuri Grin[5], Alexandra Zevalkink[3*], and Krzysztof T. Wojciechowski[1*]

[1] *Thermoelectric Research Laboratory, Department of Inorganic Chemistry, Faculty of Materials Science and Ceramics, AGH University of Krakow, Mickiewicza Ave. 30, 30-059 Krakow, Poland*
[2] *Faculty of Physics and Applied Computer Science, AGH University of Krakow, Mickiewicza Ave. 30, 30-059 Krakow, Poland*
[3] *Department of Chemical Engineering and Materials Science, Michigan State University, East Lansing, MI 48824, USA*
[4] *Department of Inorganic Chemistry, Ivan Franko National University of Lviv, Kyryla i Mefodiya St. 6, 79005 Lviv, Ukraine*
[5] *Max-Planck-Institut für Chemische Physik fester Stoffe, Nöthnitzer Str. 40, 01187 Dresden, Germany*

*E-mails: alexzev@msu.edu, wojciech@agh.edu.pl

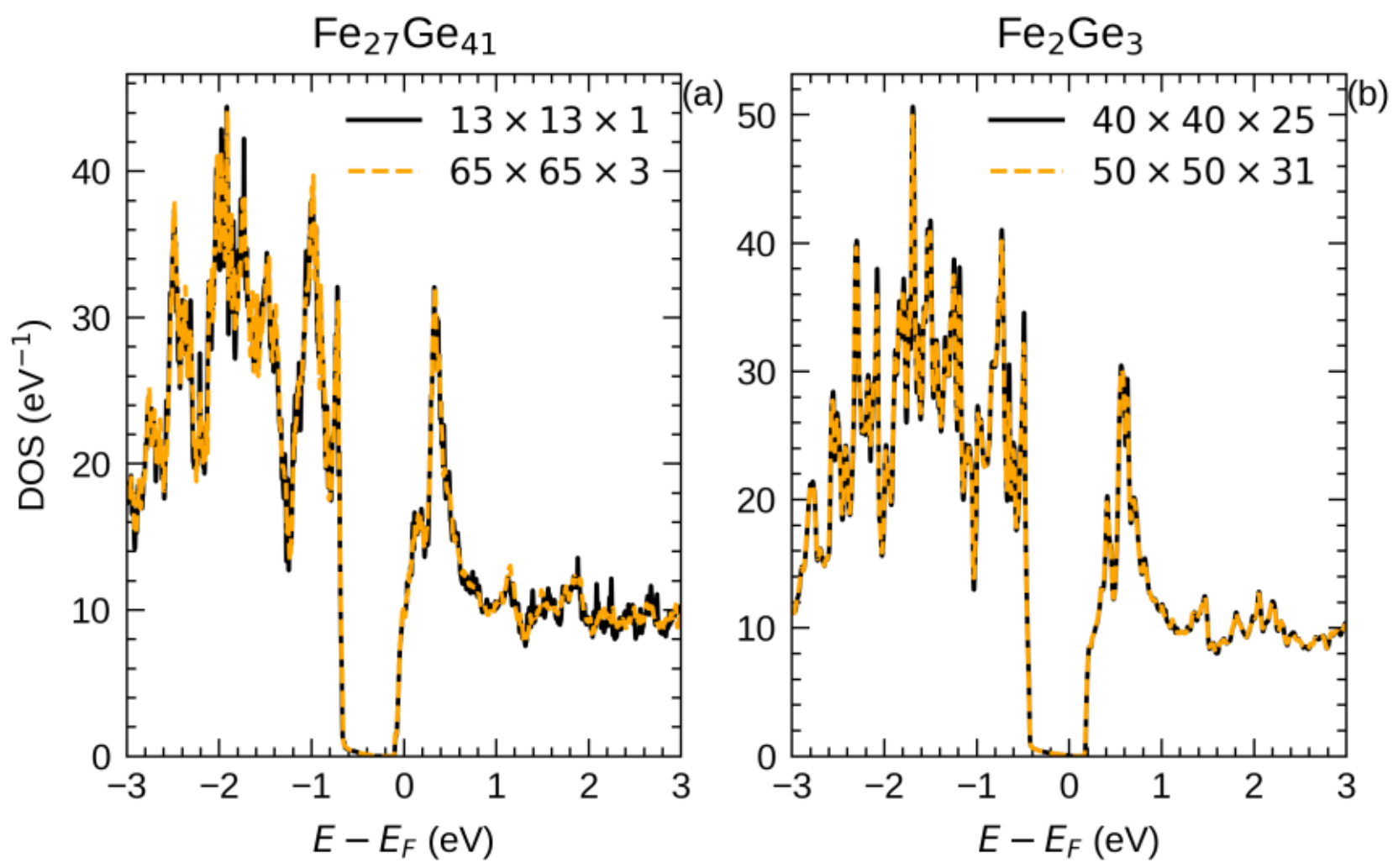

Figure S1. Density of states for optimized (a) $Fe_{27}Ge_{41}$ and (b) Fe_2Ge_3 calculated with different k-point grids. Obtained results show no meaningful differences between different k-space sampling.

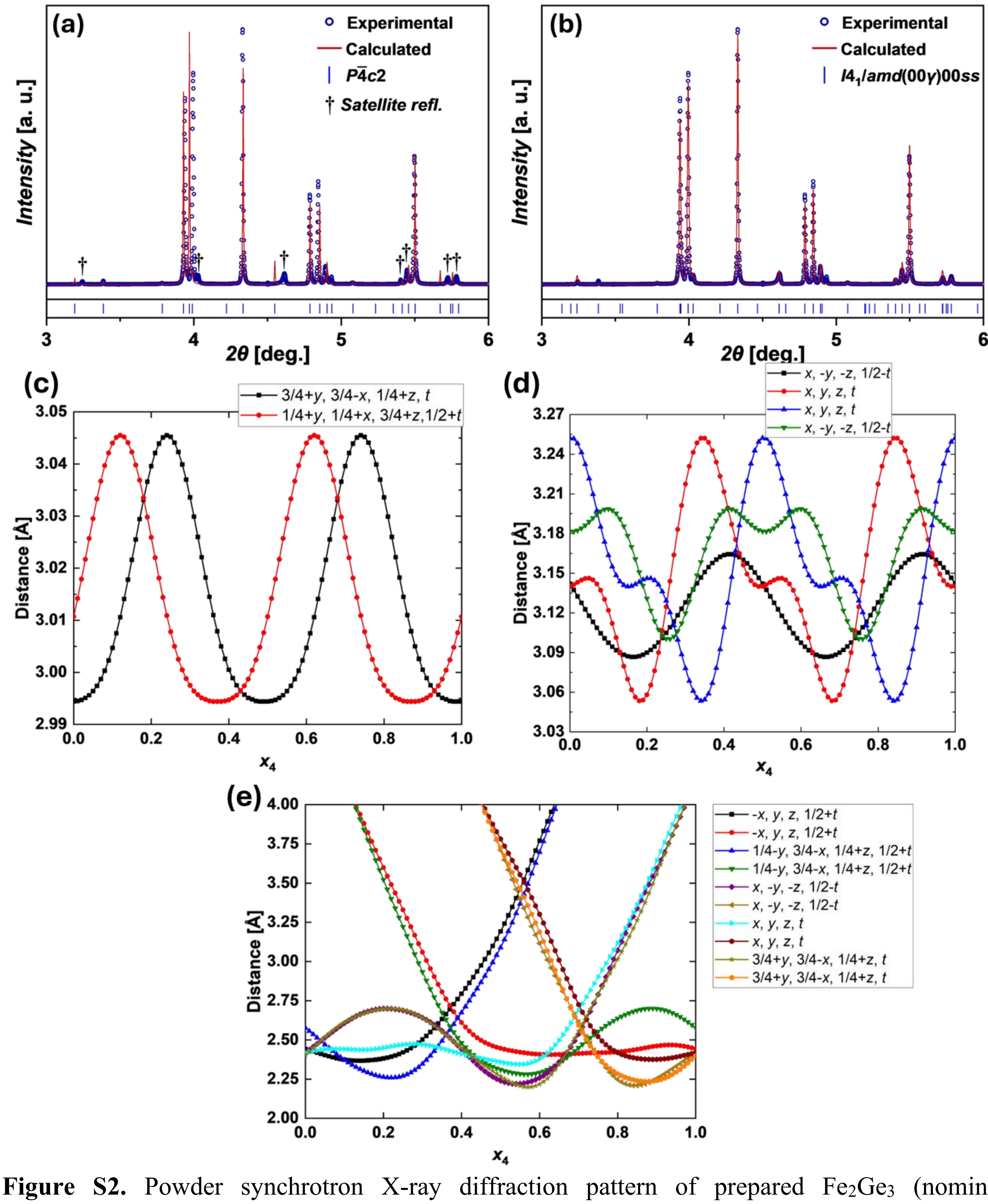

Figure S2. Powder synchrotron X-ray diffraction pattern of prepared Fe_2Ge_3 (nominal composition) sample with refinement results for commensurate structure in the space group *P*-4*c*2 with marked satellite reflections (a) and for the incommensurately modulated structure in the superspace group $I4_1/amd(00\gamma)00ss$ (b). Interatomic distances Fe-Fe (c), Ge-Ge (d), and Fe-Ge (e) as a function of x_4.

Table S1. Positional modulation wave components for Fe and Ge atoms in the incommensurately modulated structure of $FeGe_{1.52}$

U	x_4
Fe substructure	
$U_1 = \sin(2x_4)_z$	0.011(3)
$U_2 = \sin(4x_4)_z$	-0.002(8)
Ge substructure	
$U_1 = \cos(x_4)_x + \sin(x_4)_y$	-0.107(1)
$U_2 = \sin(x_4)_x + \cos(x_4)_y$	-0.022(3)
$U_3 = \cos(3x_4)_x + \sin(3x_4)_y$	-0.002(4)
$U_4 = \sin(3x_4)_x + \cos(3x_4)_y$	0.002(4)
$U_5 = \cos(4x_4)_z$	-0.008(7)
$U_6 = \sin(4x_4)_z$	-0.002(4)

Table S2. Atomic coordinates and isotropic displacement parameters for commensurate approximant structure of $Fe_{27}Ge_{41}$

Site	Wyckoff position	x	y	z	B_{iso}, Å^2
Fe1	4*h*	0	1/2	0.0275(2)	0.66(3)
Fe2	4*h*	0	1/2	0.0651(2)	0.64(3)
Fe3	4*h*	0	1/2	0.1020(2)	0.66(3)
Fe4	4*h*	0	1/2	0.1390(2)	0.65(3)
Fe5	4*h*	0	1/2	0.1755(2)	0.65(3)
Fe6	4*h*	0	1/2	0.2132(2)	0.64(3)
Fe7	4*h*	0	1/2	0.5091(2)	0.65(3)
Fe8	4*h*	0	1/2	0.5465(2)	0.66(3)
Fe9	4*h*	0	1/2	0.5835(2)	0.65(3)
Fe10	4*h*	0	1/2	0.6206(2)	0.65(3)
Fe11	4*h*	0	1/2	0.6567(2)	0.65(3)
Fe12	4*h*	0	1/2	0.6949(2)	0.65(3)
Fe13	4*h*	0	1/2	0.7318(2)	0.65(3)
Fe14	4*e*	0	0	0.0374(2)	0.66(3)
Fe15	4*e*	0	0	0.0738(2)	0.66(3)
Fe16	4*e*	0	0	0.1108(2)	0.64(3)
Fe17	4*e*	0	0	0.1480(2)	0.65(3)
Fe18	4*e*	0	0	0.1856(2)	0.66(3)
Fe19	4*e*	0	0	0.2216(2)	0.65(3)
Fe20	4*e*	0	0	0.2593(2)	0.65(3)
Fe21	4*e*	0	0	0.2960(2)	0.65(3)
Fe22	4*e*	0	0	0.3334(2)	0.66(3)
Fe23	4*e*	0	0	0.3698(2)	0.64(3)
Fe24	4*e*	0	0	0.4082(2)	0.66(3)
Fe25	4*e*	0	0	0.4440(2)	0.66(3)
Fe26	4*e*	0	0	0.4814(2)	0.65(3)
Fe27	2*c*	0	1/2	0.2500(2)	0.68(3)
Fe28	2*a*	0	0	0	0.66(3)

Ge1	8*i*	0.155(2)	0.209(2)	0.12652(7)	0.74(3)
Ge2	8*i*	0.156(2)	0.239(2)	0.27399(7)	0.73(3)
Ge3	8*i*	0.158(2)	0.202(2)	0.20219(6)	0.72(3)
Ge4	8*i*	0.162(2)	0.308(2)	0.56657(7)	0.71(3)
Ge5	8*i*	0.166(2)	0.196(2)	0.05447(7)	0.71(3)
Ge6	8*i*	0.215(2)	0.342(2)	0.00676(7)	0.70(3)
Ge7	8*i*	0.238(2)	0.354(2)	0.15222(7)	0.73(3)
Ge8	8*i*	0.260(2)	0.641(2)	0.26321(7)	0.75(3)
Ge9	8*i*	0.267(2)	0.347(2)	0.07740(7)	0.74(3)
Ge10	8*i*	0.270(2)	0.158(2)	0.61756(7)	0.73(3)
Ge11	8*i*	0.278(2)	0.658(2)	0.18820(6)	0.74(3)
Ge12	8*i*	0.297(2)	0.154(2)	0.54466(7)	0.75(3)
Ge13	8*i*	0.328(2)	0.180(2)	0.10178(7)	0.73(3)
Ge14	8*i*	0.329(2)	0.173(2)	0.17632(7)	0.72(3)
Ge15	8*i*	0.331(2)	0.313(2)	0.66579(6)	0.70(3)
Ge16	8*i*	0.335(2)	0.314(2)	0.51705(7)	0.72(3)
Ge17	8*i*	0.340(2)	0.200(2)	0.03008(7)	0.68(3)
Ge18	8*i*	0.376(2)	0.261(2)	0.58870(6)	0.78(3)
Ge19	8*i*	0.660(2)	0.191(2)	0.21330(7)	0.73(3)
Ge20	8*i*	0.672(2)	0.190(2)	0.14034(7)	0.71(3)
Ge21	4*f*	0.325(2)	0.175(2)	1/4	0.70(3)

Table S3. Atomic coordinates for the optimized structure of Fe_2Ge_3

Site	Wyckoff position	*x*	*y*	*z*
Fe1	4*i*	0	0.5	0.124265
Fe2	2*b*	0.5	0.5	0.25
Fe3	2*c*	0	0	0
Ge1	8*j*	0.656362	0.220759	0.084505
Ge2	4*f*	0.178547	0.178547	0.25

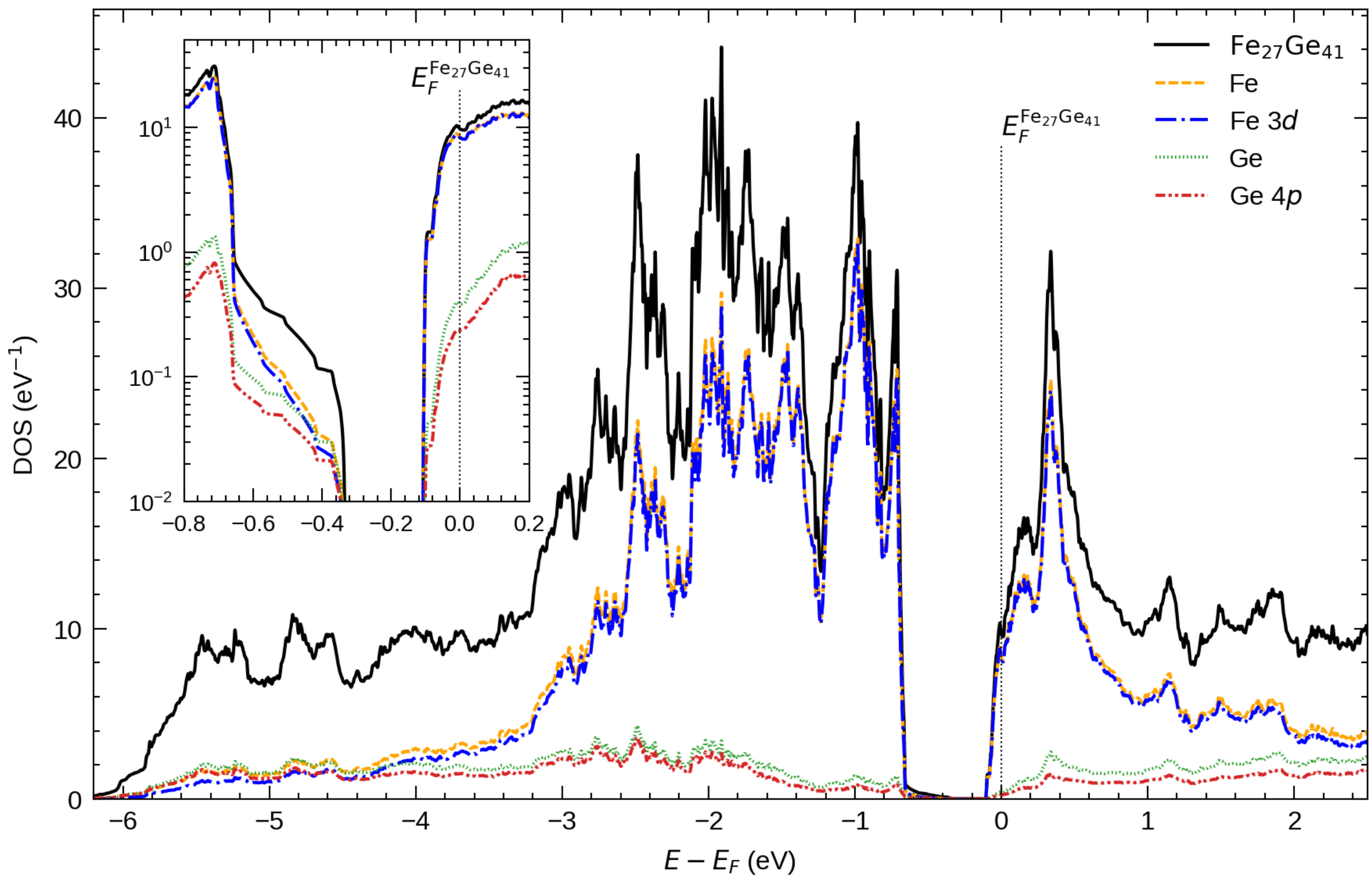

Figure S3. Electronic density of states for $Fe_{27}Ge_{41}$ with atomic contribution with the inset showing magnification in the energy range close to the band gap.

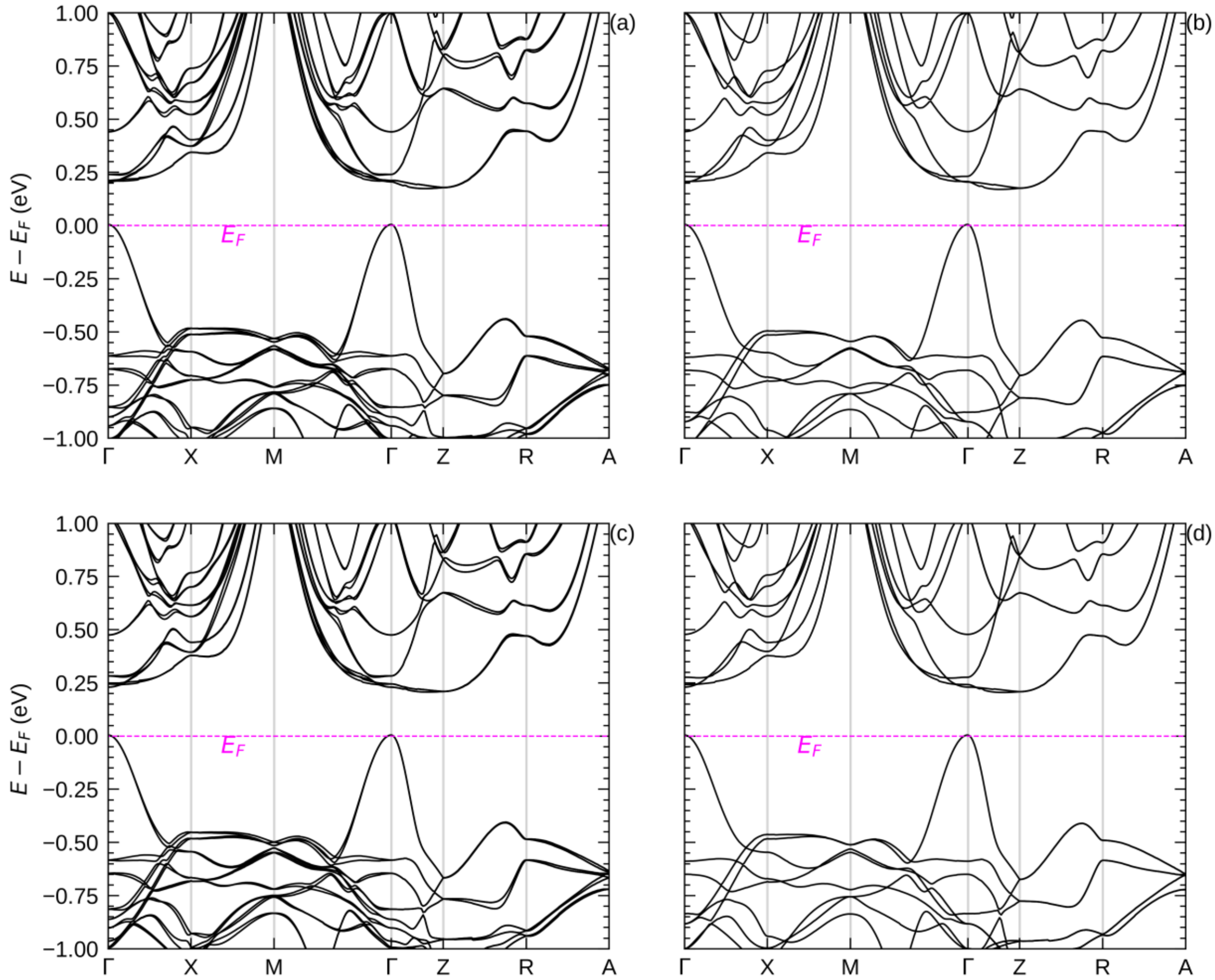

Figure S4. Electronic dispersion relation for optimized Fe_2Ge_3 with (a) and without (b) spin-orbit coupling interaction, as well as for the Fe_2Ge_3 with experimental crystal structure with (c) and without (d) spin-orbit coupling interaction. The primary difference between calculations for both structures is the slightly larger band gap of 0.2 eV in the case of the experimental structure.

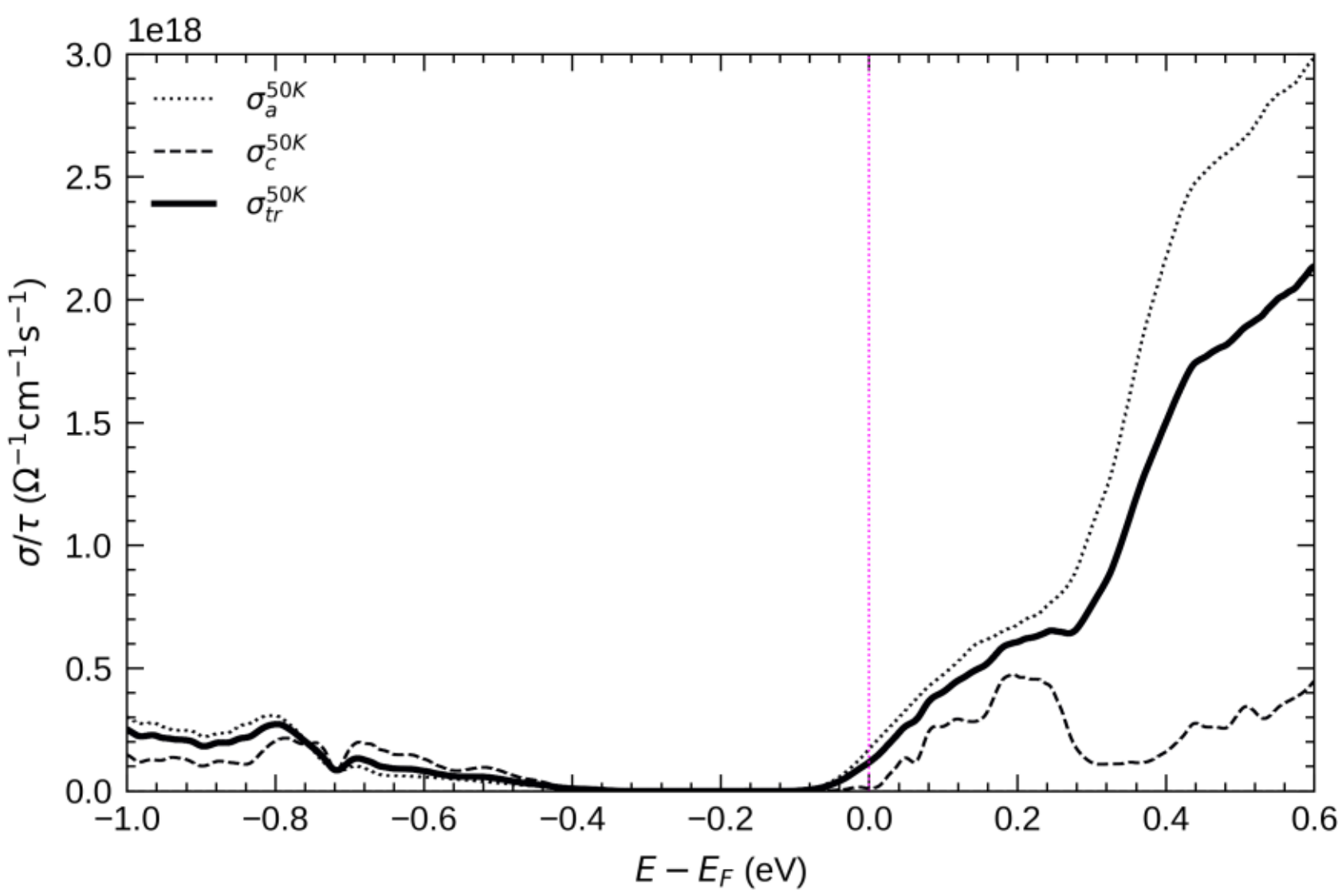

Figure S5. Energy-dependent electrical conductivity σ over relaxation time τ for $Fe_{27}Ge_{41}$ at 50 K as a function of the chemical potential μ.

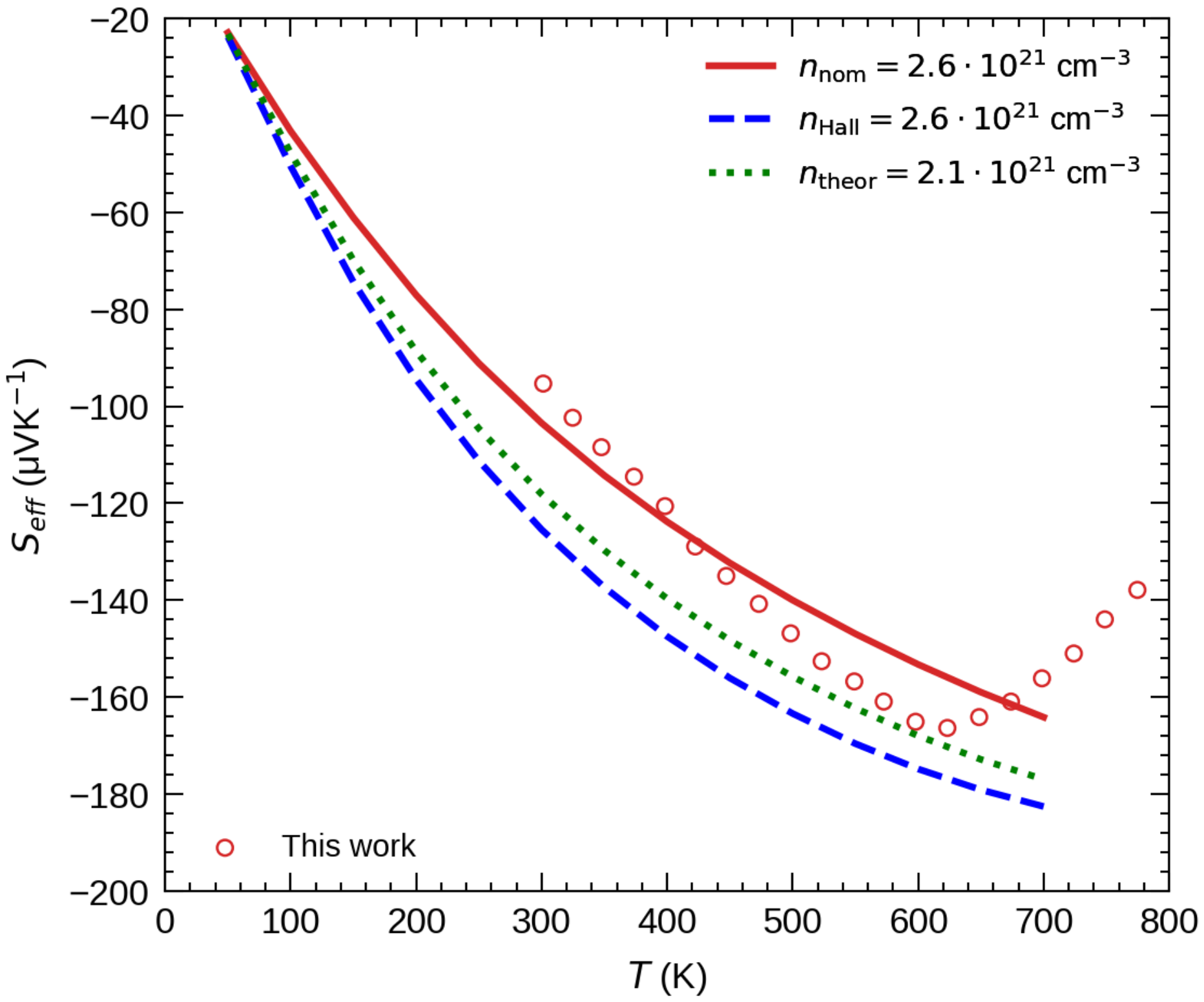

Figure S6. Temperature-dependent effective thermopower for $Fe_{27}Ge_{41}$ calculated using reduced band gap of 30 meV. Even for such strong gap reduction, the onset of bipolar effect is not observed.

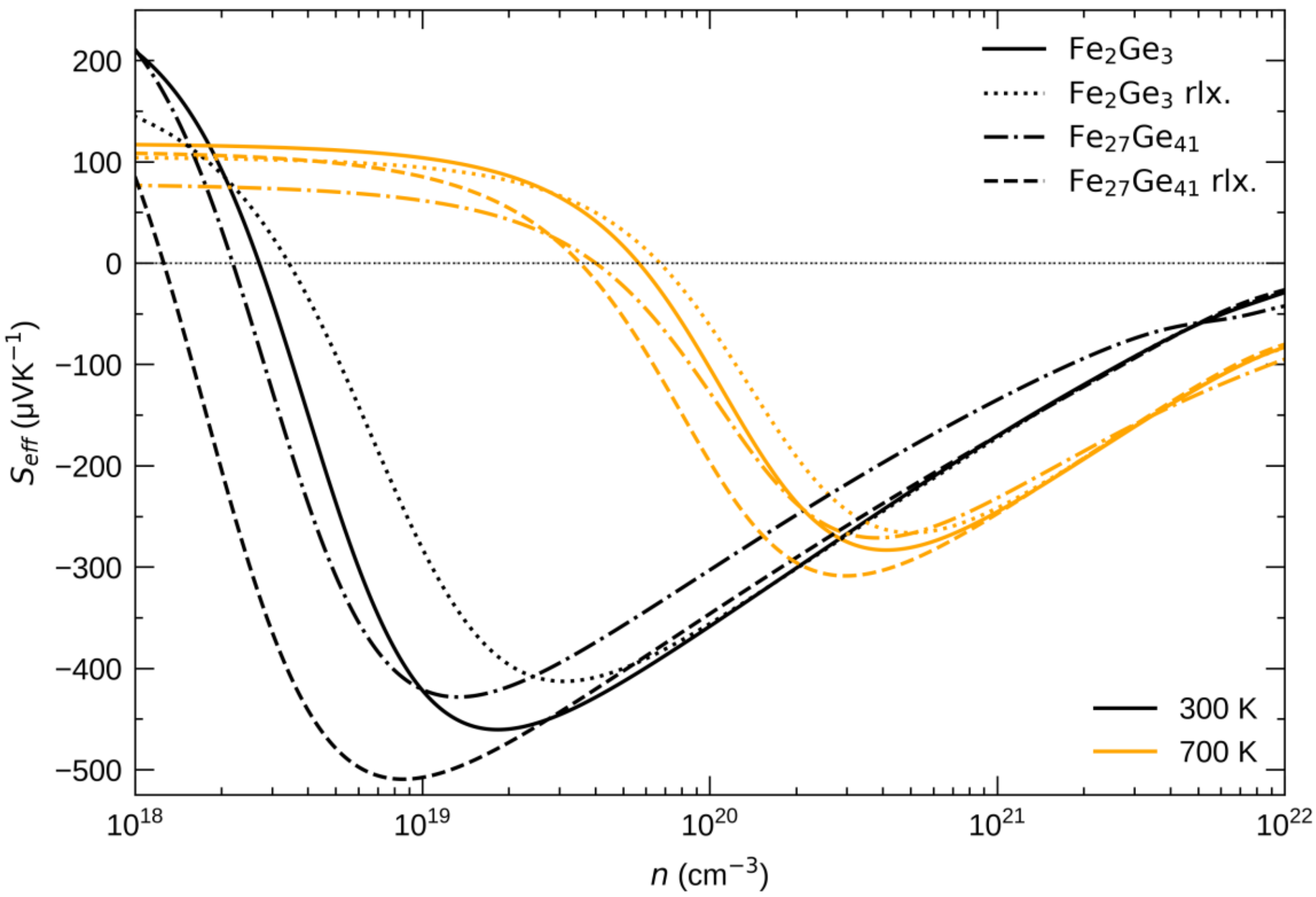

Figure S7. Calculated n-type Seebeck coefficient at T = 300 and 700 K as a function of carrier concentration for Fe_2Ge_3 and $Fe_{27}Ge_{41}$ before and after structural relaxation.

Details of the Kane band model calculations

To evaluate the density-of-states effective mass, we employed solutions of the Boltzmann transport equation. The calculations were carried out within the Kane band model approximation. In this framework, the Seebeck coefficient and carrier concentration are given by the following expressions [1,2]:

$$S = -\frac{k_B}{e}\left[\frac{I^1_{r+1,2}(\eta,\beta)}{I^0_{r+1,2}(\eta,\beta)} - \eta\right] \tag{S1}$$

$$n = \frac{\left(2m^* k_B T\right)^{3/2}}{3\pi^2\hbar^3} I^0_{3/2,0}(\eta,\beta) \tag{S2}$$

Here k_B is the Boltzmann constant, e is the charge of electron, r denotes the scattering parameter, η is the reduced chemical potential ($\eta = \mu / k_0 T$); β is the parameter in model determining non-parabolic shape of the dispersion curve ($\beta = k_B T / \varepsilon_g$, where E_g is the bandgap). $I^m_{n,k}(\eta,\beta)$ is two-parametric Fermi integrals, which can be calculated using the following expression:

$$I^m_{n,k}(\eta,\beta) = \int_0^\infty \left(-\frac{df}{dx}\right) \frac{x^m (x+\beta x^2)^n \, dx}{(1+2\beta x)^k} \tag{S3}$$

where, m denotes the power of the kinetic energy term x. It determines how strongly states at higher energy contribute to the integral and is directly related to the dimensionality of the electronic density of states. n is the exponent of the shifted term (x+β). This factor appears when the transport quantity requires an additional energy weighting. k specifies the order of the Fermi occupation function in the denominator. The case k=1 reproduces the conventional Fermi-Dirac integral, while k>1 corresponds to a further generalized form used for multi-particle statistics or advanced transport models.

In the initial stage of the analysis, the reduced chemical potential η was evaluated from the measured Seebeck coefficient S, assuming acoustic phonon scattering to be the dominant carrier relaxation mechanism. Subsequently, the density-of-states effective mass m* was extracted by combining the experimental Hall carrier concentration nH with the obtained values of η, together with the Fermi-Dirac integrals *Im,n,k(η,β)* calculated using Equation S3.

Details of the Lorenz number evaluation

The Lorenz number required to evaluate the electronic contribution to the total thermal conductivity was determined using two approaches: the parabolic band approximation and the Kane band model. For the parabolic band case, the Lorenz number was obtained using the

expression proposed by Kim *et al.* [3]. In turn, within the Single Kane band model, it was evaluated according to the following relation:

$$L_0(\eta,\beta)=\left(\frac{k_B}{e}\right)^2\left[\frac{I^2_{r+1,2}(\eta,\beta)}{I^0_{r+1,2}(\eta,\beta)}-\frac{I^1_{r+1,2}(\eta,\beta)}{I^0_{r+1,2}(\eta,\beta)}\right]. \quad \text{(S4)}$$

Debye-Callaway calculations

The lattice component of the thermal conductivity in polycrystalline systems may be estimated using the relation given in Refs. [4-5]:

$$\kappa_L=\frac{1}{3}\int_0^{\omega_D} c_V(\omega)v_S(\omega)^2\tau(\omega)d\omega \quad \text{(S5)}$$

where $c_V(\omega)$, $v_s(\omega)$, and $\tau(\omega)$ denote the specific heat, sound velocity, and phonon relaxation time as functions of frequency, respectively. The integration is carried out between zero and the Debye cutoff frequency ω_D. For temperatures exceeding the Debye temperature T_D, and assuming that the phase and group sound velocities are equivalent, the specific heat can be expressed as:

$$c_V(\omega)=\frac{3k_B\omega^2}{2\pi^2 v_s^3} \quad \text{(S6)}$$

The phonon relaxation time can be evaluated by considering the combined effect of relevant phonon-scattering processes, which are accounted for using Matthiessen's rule:

$$\tau^{-1}=\tau_U^{-1}+\tau_{GB}^{-1}+\tau_{PD}^{-1} \quad \text{(S7)}$$

The term τ_U refers to the phonon-phonon Umklapp scattering component, and τ_{GB} denotes the scattering at grain boundaries, as defined in Eqs. 8-9.

$$\tau_U(\omega)=\frac{\left(6\pi^2\right)^{1/3}}{2}\frac{Mv_s^3}{k_B V^{1/3}\gamma^2\omega^2 T} \quad \text{(S8)}$$

$$\tau_{GB}(\omega)=\frac{L}{v_s} \quad \text{(S9)}$$

where $t=\hbar\omega/(k_B T)$ and L is the grain size.

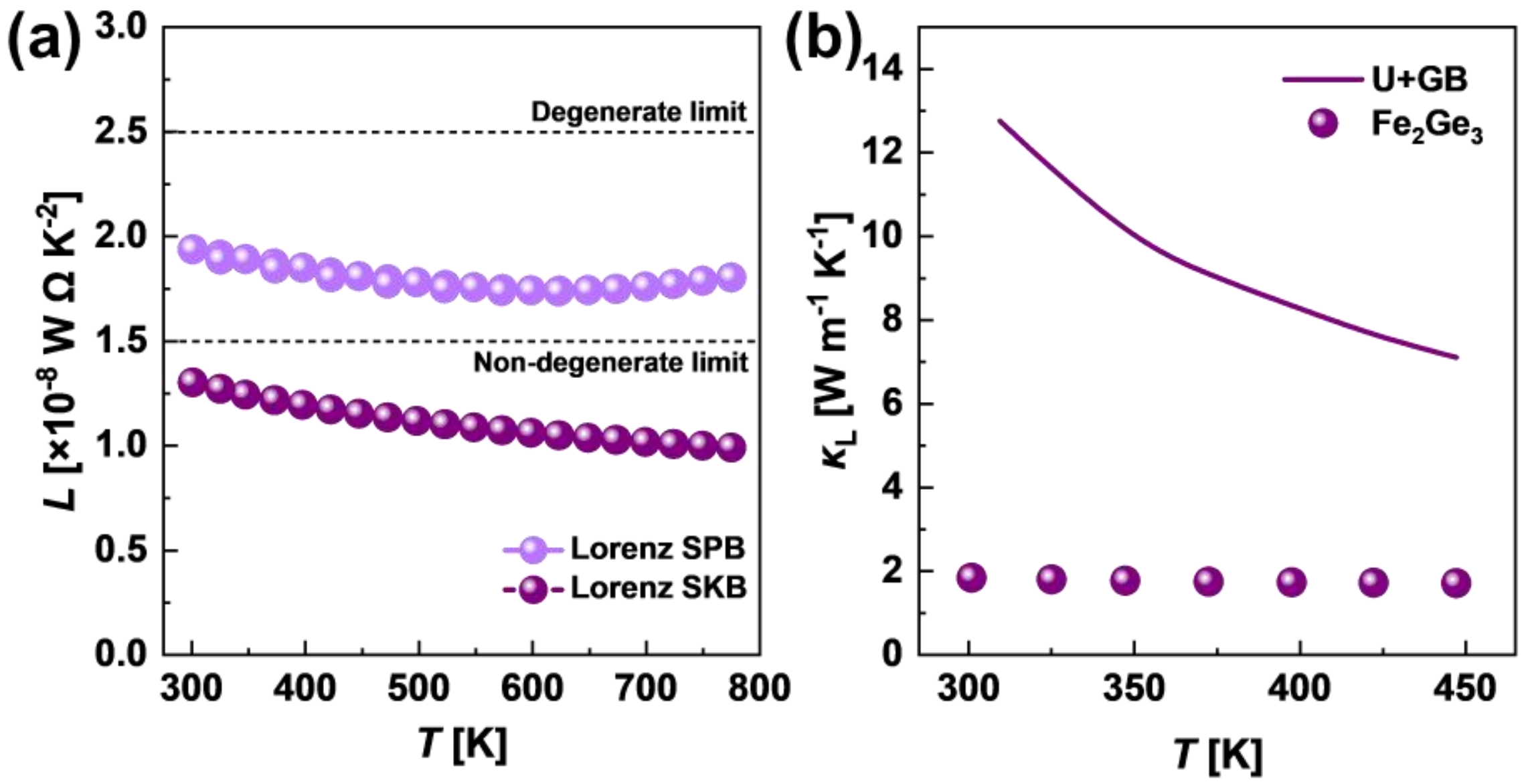

Figure S8. (a) Lorenz numbers of Fe_2Ge_3 materials calculated using a single parabolic band model (approximated as L=1.5+exp(-|S|/116)) and a single Kane band model. (b) Results of the Debye–Callaway model calculations of the lattice thermal conductivity (solid line) and the experimentally derived lattice thermal conductivity of Fe_2Ge_3, determined from thermal diffusivity measurements (LFA) and electronic thermal conductivity evaluated using the Wiedemann-Franz law.

Details of weighted mobility calculations

Weighted mobility μ_w was calculated using the following equation [4]:

$$\mu_W = \frac{3h^3}{8\pi e\rho\left(2m_e k_B T\right)^{3/2}}\left[\frac{\exp\left[\frac{|\alpha|}{k_B/e}-2\right]}{1+\exp\left[-5\left(\frac{|\alpha|}{k_B/e}-1\right)\right]}+\frac{\frac{3}{\pi^2}\frac{|\alpha|}{k_B/e}}{1+\exp\left[5\left(\frac{|\alpha|}{k_B/e}-1\right)\right]}\right] \tag{S9}$$

Details of elastic property calculations

The bulk modulus was calculated using the following equation [5]:

$$B = \rho\left(\upsilon_L^2 - \frac{4}{3}\upsilon_T^2\right) \tag{S10}$$

where ρ is the material density.

The shear modulus was calculated as:

$$G = \upsilon_T^2\rho \tag{S11}$$

The Young's modulus is calculated as:

$$E = \frac{9BG}{3B+G} \tag{S12}$$

The Poisson's ratio is calculated as:

$$\nu = \frac{E - 2G}{2G} \tag{S13}$$

The Debye temperatures were calculated using the following expression [6]:

$$\Theta_D = \frac{h}{k_B}\left[\frac{3n}{4\pi}\left(\frac{N_A \rho}{M}\right)\right]^{1/3} v_m \tag{S14}$$

where h is Planck's constant, N_A is Avogadro's number, M is the molecular weight, n is the number of atoms in the molecule, and v_m is the averaged wave velocity integrated over several crystal directions [7]:

$$v_m = \left[\frac{1}{3}\left(\frac{2}{v_t^3} + \frac{1}{v_l^3}\right)\right]^{-1/3} \tag{S15}$$

where v_l and v_t are the longitudinal and transverse sound velocities, respectively. Grüneisen parameters γ were calculated using the following formula [8]:

$$\gamma = \frac{3}{2}\left(\frac{1+\nu}{2-3\nu}\right) \tag{S16}$$

Details of the thermal transport property calculations

The phonon mean free paths were calculated by [8]:

$$l_{ph} = \frac{3\kappa_L}{C_V v_m} \tag{S17}$$

Considering the ultrasonic data, the lattice thermal conductivity was also calculated as follows [8]:

$$\kappa_L = K\frac{\bar{M}\Theta_D^3\delta}{\gamma^2 n^{\frac{2}{3}} T} \tag{S18}$$

Here n is the number of atoms in the primitive unit cell, d^3 is the volume per atom, Θ_D is the Debye temperature, $\overline{M}$ is the average mass of the atoms in the crystal, and K is a collection of physical constants ($K \approx 3.1\times10^{-6}$ if κ_L is in $Wm^{-1}K^{-1}$, $\overline{M}$ in amu, and δ in Angstroms).

According to Cahill's formulation based on the maximum phonon scattering approach, the glass limit for the thermal conductivity κ_{glass} was estimated by:

$$\kappa_{glass} = \frac{1}{2}\left(\frac{\pi}{6}\right)^{1/3} k_B V^{-2/3}\left(2v_t + v_l\right) \tag{S19}$$

where V is the average volume per atom calculated from the refined lattice parameters.

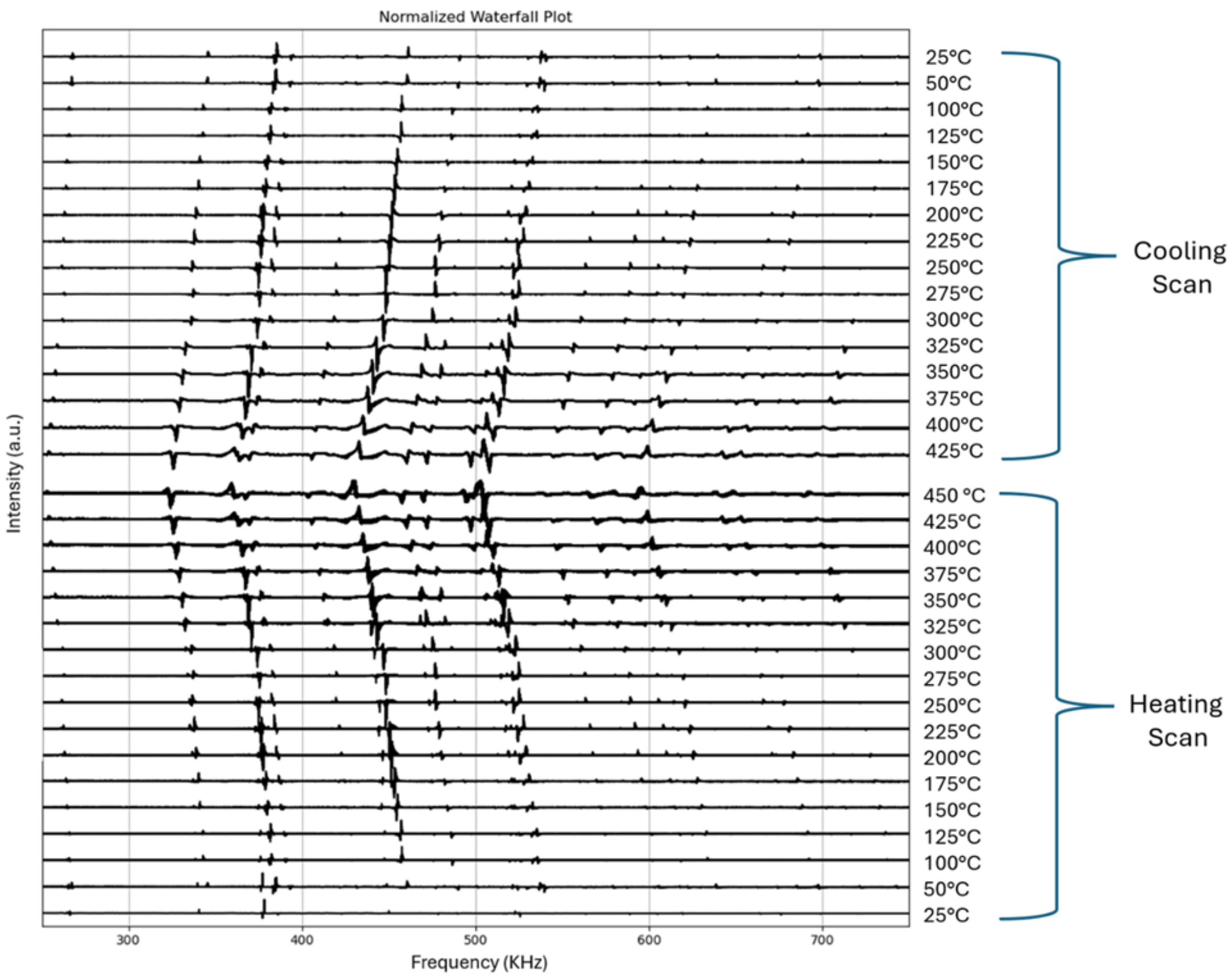

Figure S9. Normalized waterfall plot showing the variation of resonance frequency with temperature during sequential heating (25°C to 450°C) and cooling (425°C to 25°C) scans. The frequency decreases progressively with increasing temperature during the heating cycle, while the opposite trend is observed during cooling. The reversible shift in frequency indicates temperature-dependent structural or elastic changes in the material.

References

[1] Y.I. Ravich, B.A. Efimova, I.A. Smirnov, Semiconducting Lead Chalcogenides, Springer US, 1970.

[2] B.M. Askerov, Electron Transport Phenomena in Semiconductors, 1994.

[3] H.S. Kim, Z.M. Gibbs, Y. Tang, H. Wang, G.J. Snyder, Characterization of Lorenz number with Seebeck coefficient measurement, APL Mater. 3 (2015) 041506.

[4] Snyder, G. J.; Snyder, A. H.; Wood, M.; Gurunathan, R.; Snyder, B. H.; Niu, C. Weighted Mobility. Advanced Materials 2020, 32 (25), 2001537.

[5] E.P. Papadakis, E.P. Papdakis, C.A. Stickels, R.C. Innes, An Ultrasonic Technique for Measuring the Elastic Constants of Small Samples, SAE Trans. 104 (1995) 830–837.

[6] O.L. Anderson, A simplified method for calculating the Debye temperature from elastic constants, J. Phys. Chem. Solids. 24 (1963) 909–917.

[7] E. Isotta, W. Peng, A. Balodhi, A. Zevalkink, Elastic moduli: a tool for understanding chemical bonding and thermal transport in thermoelectric materials. Angew. Chem. Int. Ed., 135 (2023) e202213649.

[8] H. Xie, S. Hao, S. Cai, T.P. Bailey, C. Uher, C. Wolverton, V.P. Dravid, M.G. Kanatzidis, Ultralow thermal conductivity in diamondoid lattices: high thermoelectric performance in chalcopyrite $Cu_{0.8+y}Ag_{0.2}In_{1-y}Te_2$, Energy Environ. Sci. 13 (2020) 3693–3705.
